# Analysis of novel zinc-binding proteins in the cell wall of *Corynebacterium diphtheriae*

**DOI:** 10.1128/jb.00239-25

**Published:** 2025-08-18

**Authors:** Eric D. Peng, Lindsey R. Lyman, Michael P. Schmitt

**Affiliations:** 1 Laboratory of Respiratory and Special Pathogens, Division of Bacterial, Parasitic, and Allergenic Products, Center for Biologics Evaluation and Research, Food and Drug Administration 4137 , Silver Spring, Maryland, USA; University of Illinois Chicago, Chicago, Illinois, USA

**Keywords:** *Corynebacterium*, zinc, sortase

## Abstract

Zinc is a critical nutrient for all living organisms, including bacterial pathogens such as *Corynebacterium diphtheriae*, the causative agent of the severe human respiratory disease diphtheria. As such, zinc acquisition is essential for many pathogens to cause disease. We previously showed that the zinc-regulated ABC transporter encoded by the *znu* locus is one of several zinc uptake systems that support the growth of *C. diphtheriae* in zinc-limited medium. In this study, we examine the function and cellular localization of components encoded by the *znu* gene cluster, which includes the ZnuABC transporter and the novel membrane and cell wall proteins ZnuE, ZnuF, and ZnuG. Deletion of *znuE* results in reduced growth in zinc-limited media, suggesting a role in zinc uptake. While the growth of *znuF* and *znuG* mutants was not affected in zinc-limited medium, deletion of these two genes in a mutant also lacking the *znuABC* transporter restored growth to wild-type levels, suggesting that ZnuF and ZnuG exert an unusual modulating effect on zinc import. ZnuE, ZnuF, and ZnuG possess a unique motif that is associated with Zn binding as demonstrated by thermal shift assays, targeted mutagenesis, and structural analysis. Although ZnuF and ZnuG are both present in the cell wall, only ZnuG contains a sortase recognition signal that is critical for localizing proteins to the cell wall. Furthermore, ZnuF localization does not require any of the six known sortase enzymes in *C. diphtheriae*, suggesting a novel localization mechanism.

**IMPORTANCE:**

Zinc is a critical nutrient required by many bacterial pathogens. While the function of multiple zinc importer systems has been previously characterized in *Corynebacterium diphtheriae*, the transporter encoded by the *znu* gene cluster includes components not found in other metal transport systems. In this report, we examined the roles of three components of the *znu* gene cluster, ZnuE, ZnuF, and ZnuG, and show that these proteins all possess a putative zinc-binding domain and have varying effects on growth in zinc-limited medium. Additionally, ZnuF uses a novel mechanism for cell wall localization. This study further expands our understanding of *C. diphtheriae* zinc import and points to a potentially novel mechanism for the localization of cell wall proteins.

## INTRODUCTION

Zinc is a critical nutrient for bacterial pathogens such as *Corynebacterium diphtheriae*, the etiological agent of the respiratory disease diphtheria and cutaneous ulcers. To cause disease, *C. diphtheriae* must encode proteins and other factors necessary for the colonization of its host. These include not only virulence factors, such as diphtheria toxin, and adhesion mechanisms, such as pilin proteins, but also systems to acquire essential nutrition. Zinc is an essential element with structural and catalytic roles in proteins, and it has been reported that 5%–6% of bacterial proteins require zinc as a cofactor (1). Despite an abundance of zinc within the human host, zinc is mostly bound to host proteins and sequestered during inflammation by calprotectin and other mechanisms (2–4). Host restriction of metals that are important for biological processes, a feature known as nutritional immunity, functions as a nonspecific defense against invading pathogens (4).

To overcome nutritional immunity, bacterial pathogens encode one or more high-affinity import systems. Mechanisms for zinc import include ATP-binding cassette transporters (or ABC transporters), the use of zinc scavenging molecules (metallophores), and ZIP/ZupT family transporters (4). *Streptococcus pneumoniae* uses a ZnuABC/AdcABC family transporter with two distinct substrate-binding proteins (SBPs) for zinc acquisition (5); *Staphylococcus aureus* uses an Adc family transporter and produces a metallophore, staphylopine (StP), a zinc scavenging small molecule (6). *Clostridioides difficile* utilizes a ZupT family transporter and other factors for countering host zinc restriction (7). Previously, we found that *C. diphtheriae* encodes several ABC transporters and potentially uses zinc scavenging molecules for zinc acquisition (8). We identified six different loci that support growth under zinc limitation: *iut*, *mnt*, *nik1*, *nik2*, *sid*, and *znu*. The *C. diphtheriae iut* gene cluster encodes an ABC transporter with two separately expressed SBPs, IutA and IutE (9). We previously reported that the IutA-D transport system is involved in zinc uptake (8), and while both IutA and IutE can bind to Zn^2+^ and Mn^2+^, they are differentially expressed in response to zinc (9–11), manganese (11), and iron (9). The reason for their differential regulation remains unclear, and a defined role for IutE in metal transport has not been determined. The *mnt* locus is a Mn-regulated ABC transporter with similarity to Mn/Zn importers and can import Zn (8, 11). The *nik1* and *nik2* loci encode ABC transporters related to uptake systems required for the import of nickel and small peptides, as well as metallophores such as StP produced by *S. aureus* (6). The *sid* genes are predicted to encode proteins involved in the biosynthesis of a small molecule (12), similar to those required for the biosynthesis of the metallophore yersiniabactin identified in *Yersinia pestis* (13) and analogous to the proteins required for *S. aureus* StP biosynthesis (6). Finally, the *znu* locus encodes an ABC transporter with a SBP (ZnuA), permease (ZnuC), an ATPase (ZnuB), and an additional membrane protein of undefined function (ZnuE) (8). In zinc-limited medium, a strain deleted of all six loci (*iut*, *mnt*, *nik1*, *nik2*, *sid*, and *znu*, designated Δ6) exhibits significantly reduced growth relative to the wild-type strain, while strains that retain any one of the six loci show enhanced growth when compared to the Δ6 strain. The *znu* gene cluster includes the *znu* locus, *znuAECB,* and an adjacent downstream locus, *znuF-znuG-dip0444-dip0445* (*znuF-445*). Gene expression studies indicate that the *znu* gene cluster is composed of two distinct operons, with promoters located upstream of *znuA* and *znuF* (10). Both promoters are repressed in response to zinc by the zinc-responsive global transcriptional regulator, Zur (10). In the absence of the other five zinc transporter loci (*iut*, *mnt*, *sid*, *nik1*, and *nik2*), the *znuAECB* locus supports wild-type levels of growth in zinc-limited medium (8).

In this study, we characterized three proteins encoded in the *znu* gene cluster: ZnuE, ZnuF, and ZnuG. We show that the proteins have different effects on the use of zinc for growth in zinc-limited medium, and that each of these proteins can bind zinc, likely through a novel zinc-binding motif. We showed that, surprisingly, both ZnuG and ZnuF are localized to the cell wall, and that ZnuF is exposed on the bacterial surface. ZnuF lacks a sortase signal in its C-terminal, which is typically required for cell wall anchoring. None of the six sortases in *C. diphtheriae* were required for the cell wall localization of ZnuF, suggesting a novel mechanism for its trafficking. This report expands our understanding of a novel zinc transport system and provides evidence for an alternative mechanism of cell wall protein localization in *C. diphtheriae*.

## RESULTS

### The *znu* gene cluster encodes two distinct zinc-regulated operons

**Fig 1:**
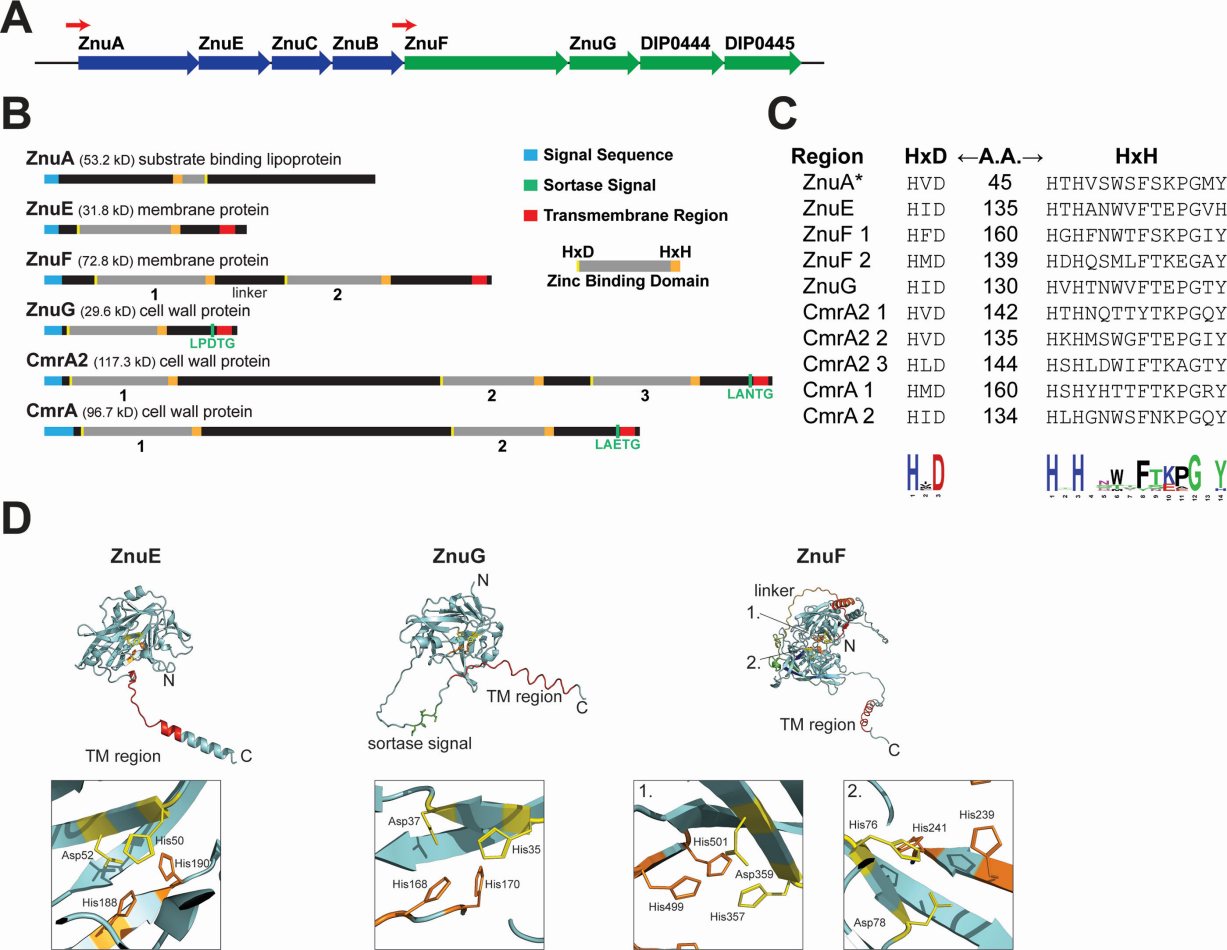
The *znu* gene cluster and conserved motif. (**A**) Gene cluster structure of the *znuAECB* and *znuF-445* loci. Red arrows indicate approximate promoter regions. (**B**) General features for proteins of interest with indicated signals: signal sequence (blue), sortase signal (green), and transmembrane region (red). The amino acid regions that encompass the conserved HxD/HxH are indicated (yellow, gray, and orange); the number under each region correlates to the number provided in panel **C**, which provides the amino acid sequence for each conserved HxD/HxH motif and the number of amino acids found between the HxD and HxH. The conserved motifs were subjected to analysis by the Multiple Em for Motif Elicitation algorithm (15) to generate the consensus sequence shown below. *In ZnuA, the HxD and HxH are in reverse order. (**D**) Protein structure predictions provided in the AlphaFold Protein Structure Database (16, 17) for ZnuE, ZnuF, and ZnuG; enlarged images show the relative locations of the conserved HxD and HxH residues. The linker region indicated in ZnuF is the region between the HxH motif of ZnuF region 1 and the HxD motif of ZnuF region 2.

The *znu* gene cluster includes the *znu* locus, which encodes the Znu ABC transporter (ZnuAECB), and the *znuF-445* locus, which includes four proteins of unknown function: ZnuF, ZnuG, DIP0444, and DIP0445 (Fig. 1A). The *znuA* (SBP)*, znuC* (ATPase)*,* and *znuB* (permease) genes in the *znu* locus encode proteins required for an ABC transporter; however, *znuE* encodes a predicted membrane protein of unknown function that is not found with other ABC-type transporters. Furthermore, ZnuE shares amino acid similarities with the ZnuF and ZnuG proteins (encoded in the *znuF-445 locus*), as well as with zinc-regulated proteins CmrA and CmrA2 (10), which are encoded elsewhere in the genome (Fig. 1B and C). The sequence similarities among these proteins are a conserved His-x-Asp and His-x-His (HxD/HxH) motif that brackets approximately 150 amino acid regions of low sequence conservation (Fig. 1C; Fig. S1). These motifs are present with varying copy numbers among these zinc-regulated proteins, with ZnuE and ZnuG each having one motif, ZnuF and CmrA having two, and CmrA2 having three (Fig. 1C). ZnuA, the SBP, also encodes an HxD/HxH motif, but in the reverse orientation and with a shorter intervening region (45 amino acids). Independent of the HxD/HxH motif, ZnuA shows a high level of similarity with other zinc-binding ZnuA-family SBPs, including a highly conserved zinc-binding site that includes three His residues (H63, H317, and H383) not associated with the HxD/HxH motif. Like most SBPs in gram-positive bacteria, ZnuA contains signals required for secretion and lipid anchoring in its N-terminal region. ZnuE and ZnuF have secretion signals and a putative membrane anchoring region that includes a C-terminal transmembrane domain and a positively charged C-terminus, suggesting these proteins are secreted but may be associated with the cell membrane. ZnuG, CmrA2, and CmrA are predicted to be cell wall proteins based on the presence of cell wall sorting signals consisting of a sortase recognition sequence, a transmembrane region, and a charged tail, which is required for covalent anchoring of proteins to the bacterial cell wall by sortase enzymes (14).

Predicted structures of the proteins ZnuE, ZnuF, and ZnuG are available in the AlphaFold Protein Structure Database (16, 17) and shown in Fig. 1D. For ZnuE and ZnuG, the HxD/HxH residues are predicted to be closely associated in the tertiary structure (Fig. 1D). The HxD/HxH residues form a pocket like those found in the ZnuA SBPs in *Escherichia coli* (18) and *Citrobacter koseri* (19), which both coordinate Zn^2+^ with three His and one Glu residue. ZnuF contains two motifs each forming a separate domain that resembles the structure of ZnuE and ZnuG; the domains are connected by an unstructured linker. Based on the structural predictions, it seems likely that these motifs function as zinc-binding domains. The last two genes in the *znu* gene cluster, *dip0444* and *dip0445*, are both predicted to encode membrane proteins. The DIP0444 protein has no significant similarity to known proteins, and DIP0445 has significant sequence homology to PorA-like proteins, which are predicted to form channels in the cell wall. The function of either of these proteins has not been determined.

### Deletion of downstream genes in the *znu* gene cluster restores wild-type growth to the *znuAECB* deletion mutant

**Fig 2:**
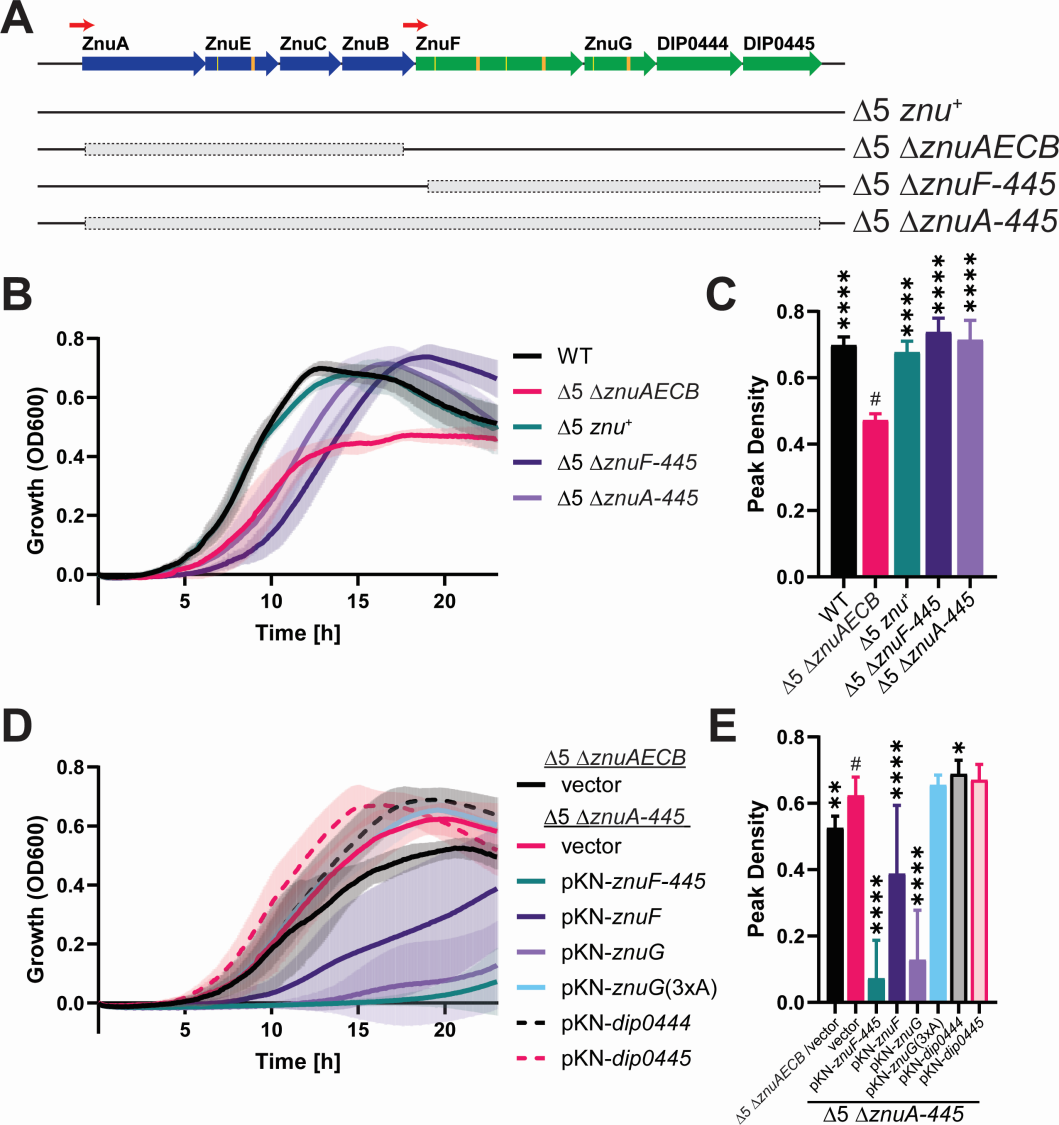
Deletion of *znuF-0445* alleviates the zinc limitation growth defect. (**A**) The *znuAECB* and *znuF-0445* gene cluster structure and genotype for the indicated strains are shown. Gray boxes indicate regions that were deleted by allelic exchange. (**B** and **D**) Growth of the indicated strains was monitored over 23 h with the OD_600_ measured at 5-min intervals. Data shown are the mean (solid line) and standard deviation (shaded area) of replicates. (**C** and **E**) The highest measured OD_600_ mean and respective standard deviation from the data in panels **B** and **D** are shown for statistical comparison. Note that the peak density for each strain occurred at different times. An ordinary one-way ANOVA Fisher’s LSD test was used to compare samples against the indicated strain (#). *P < 0.05, **P < 0.01, and ****P < 0.0001; *n* ≥ 12.

To determine whether the proteins encoded in the *znu* gene cluster are involved in zinc transport, we introduced targeted mutations into the Δ5 *znu*^+^ strain, which lacks *iut*, *mnt*, *nik1*, *nik2*, and *sid* but retains the complete *znu* gene cluster (Fig. 2A) (8). In zinc-limited medium, the Δ5 *znu*^+^ strain grows to wild-type density, while a strain lacking all six transport-associated loci, Δ5 Δ*znuAECB* (previously described as Δ6, but renamed herein for clarity) displays reduced growth (Fig. 2B and C and reference 8). To assess the function of the gene products encoded in the *znuF-445* locus, we introduced the deletion of the complete *znuF-445* operon into strain Δ5 *znu*^+^ (resulting in strain Δ5 Δ*znuF-445*) and also into the Δ5 Δ*znuAECB* strain (resulting in strain Δ5 Δ*znuA-445*) (Fig. 2A). The Δ5 Δ*znuF-445* strain showed similar peak density to that of Δ5 *znu*^+^ but an increased duration of the lag phase (Fig. 2A through C). However, the same deletion in the Δ5 Δ*znuAECB* background resulted in a greater peak cell density for the Δ5 Δ*znuA-445* strain compared to the peak cell density of Δ5 Δ*znuAECB* in zinc-limited media but also a similar increased lag phase duration. These findings suggest that one or more proteins encoded in the *znuF-445* operon result in reduced growth yield of the Δ5 Δ*znuAECB* strain since the additional deletion of *znuF-445* in the Δ5 Δ*znuAECB* strain resulted in increased peak cell density in low zinc media. Additionally, the loss of one or more components of the *znuF-445* locus appears to increase the duration of the lag phase.

To determine which genes in the *znuF-445* locus were responsible for the reduced growth observed in the Δ5 Δ*znuAECB* strain when compared to the growth of Δ5 Δ*znuA-445*, we introduced plasmids encoding genes of the *znuF-445* operon into the Δ5 Δ*znuA-445* strain. A plasmid expressing all four genes from their native promoter (pKN-*znuF-445*) in the Δ5 Δ*znuA-445* strain resulted in a severe growth defect (Fig. 2D and E). To determine which of the four genes is responsible for the growth defect, we cloned individual genes expressed from a constitutive promoter, pO5 (8, 20). Plasmids encoding either *znuF* or *znuG* resulted in reduced growth. The introduction of the cloned genes *dip0444* or *dip0445* had little effect on growth, and a function for these gene products is not known (Fig. 2D and E). ZnuF and ZnuG both possess conserved HxD/HxH motifs; for ZnuG, we substituted all three of the conserved His residues to Ala (HxD/HxH→AxD/AxA) to determine if this motif contributes to the reduced growth. A plasmid carrying mutated *znuG* [pKN-*znuG*(3xA)] did not affect the growth of the Δ5 Δ*znuA-445* strain (Fig. 2D and E), suggesting that the His residues are required for the reduced growth phenotype. The findings indicate that only ZnuG and ZnuF are required for the reduced growth phenotype and suggest that these proteins may inhibit zinc import in the Δ5 Δ*znuAECB* strain, which contains a deletion of the *znuAECB* locus. In the presence of the *znuAECB* locus (Δ5 *znu*^+^), ZnuG and ZnuF appear to have no significant impact on growth in zinc-limited media, since the Δ5 *znu*^+^ strain grows like the wild-type strain.

### ZnuE is critical for growth in zinc-limited medium

**Fig 3:**
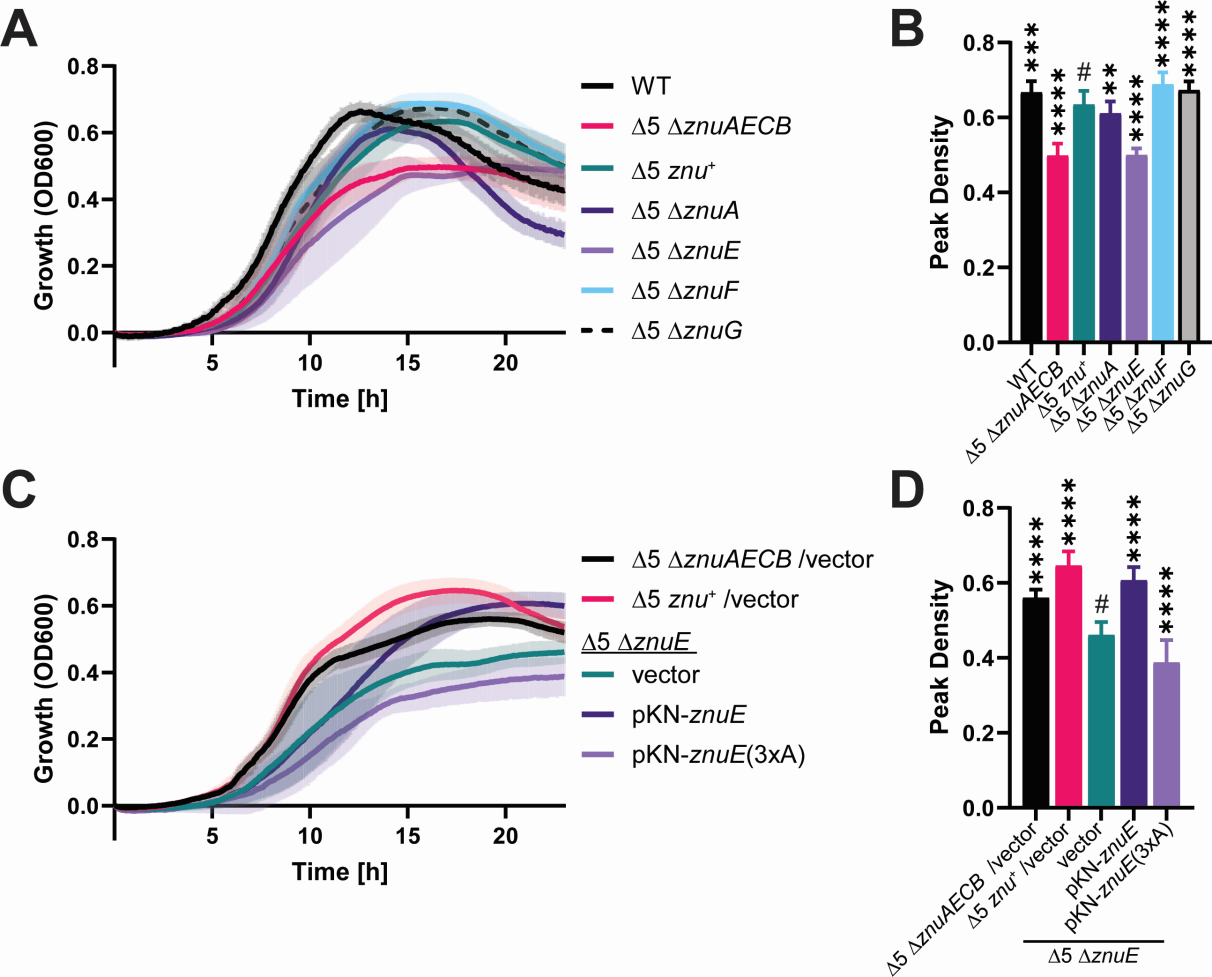
*znuE* is important for Znu transporter function. (**A** and **C**) Growth of the indicated strains was monitored over 23 h with the OD_600_ measured at 5-min intervals. Data shown are the mean (solid line) and standard deviation (shaded area). (**B** and **D**) The highest measured OD_600_ mean and respective standard deviation from the data in panels **A** and **C** are shown for statistical comparison. Note that the peak density for each strain occurred at different times. An ordinary one-way ANOVA Fisher’s LSD test was used to compare samples against the indicated strain (#). **P < 0.01, ***P < 0.001, and ****P < 0.0001; *n* ≥ 12.

The *znu* transporter locus, *znuAECB*, contains genes encoding a substrate-binding protein (*znuA*), a membrane protein of unknown function (*znuE*), an ATPase (*znuC*), and a permease (*znuB*). The protein encoded by *znuE* shares the conserved HxD/HxH motif found in ZnuF and ZnuG. To test the function of *znuA* and *znuE* in zinc import, we created in-frame deletion mutants for these two genes in the Δ5 *znu*^+^ strain: designated Δ5 Δ*znuA* and Δ5 Δ*znuE* (Fig. 3A). Based on the results observed in Fig. 2, we also created in-frame deletions of *znuF* and *znuG* in the Δ5 *znu*^+^ strain to assess their effect on zinc import. Surprisingly, the deletion of *znuA*, encoding the substrate-binding protein, only had a modest impact on growth in our test conditions; however, the deletion of *znuE* (Δ5 Δ*znuE*) resulted in growth comparable to the Δ5 Δ*znuAECB* strain, suggesting a significant decrease in zinc import function in the absence of *znuE* (Fig. 3A and B). Plasmid-encoded *znuE* was introduced into the Δ5 Δ*znuE* strain and shown to restore growth (Fig. 3C and D), confirming that the loss of *znuE* is responsible for the growth defect and suggesting that ZnuE may have a role in the transport of zinc. Consistent with results for ZnuG (Fig. 2D and E), mutating the His residues to Ala in the ZnuE HxD/HxH motif [pKN-*znuE*(3xA)] renders it unable to complement the growth defect, suggesting that the HxD/HxH motif in ZnuE is required for maximal growth in zinc-limited medium. The deletion of either *znuF* or *znuG* in the Δ5 *znu*^+^ strain did not impact growth, suggesting that neither protein is required for zinc uptake in the Δ5 *znu*^+^ strain (Fig. 3A and B). While the functions of ZnuF and ZnuG are unclear, additional studies may determine what roles these proteins have in zinc metabolism in *C. diphtheriae*.

### ZnuE and ZnuF bind Zn *in vitro*

**Fig 4:**
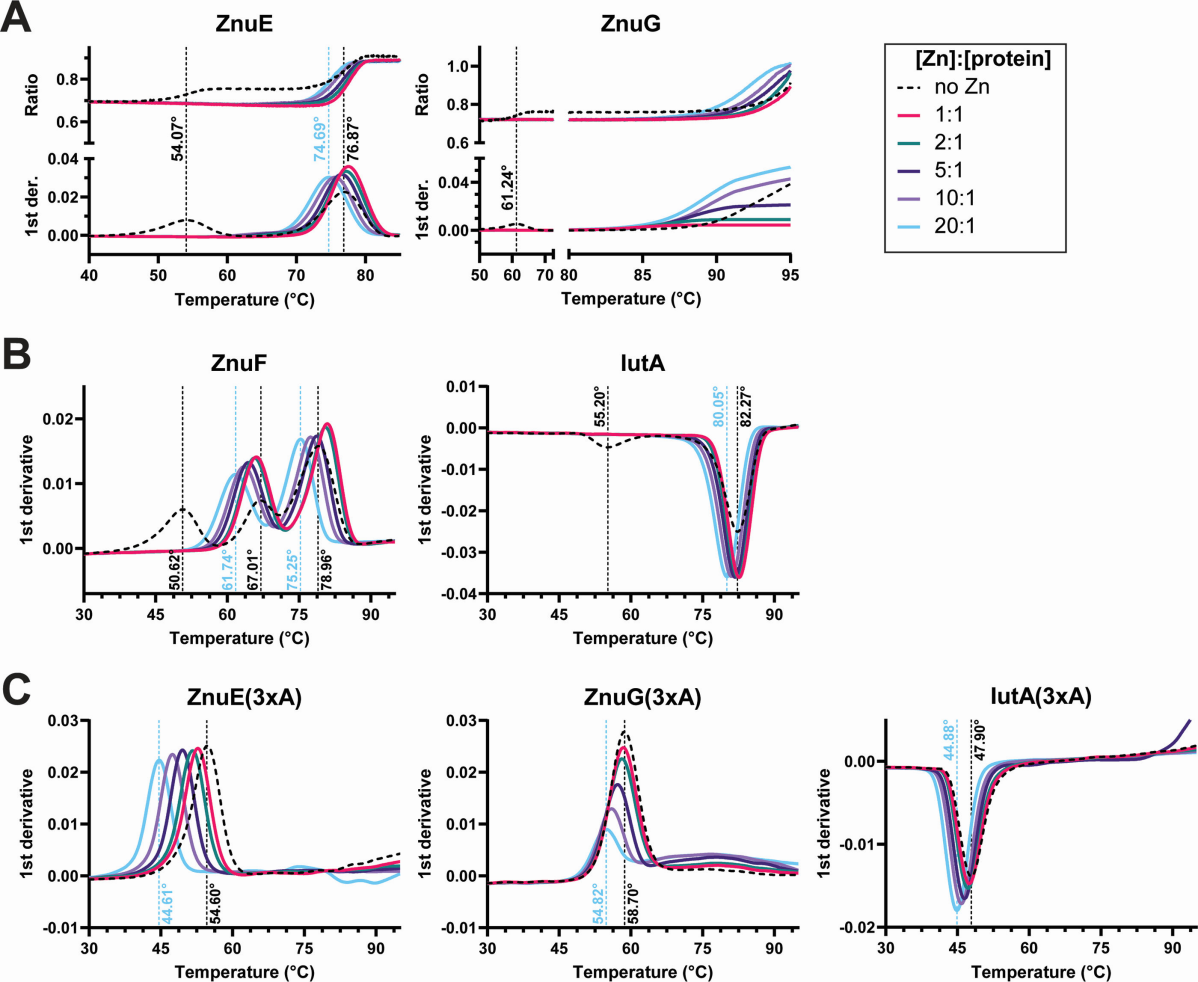
ZnuE and ZnuF bind Zn by thermal shift assay. Each indicated protein was incubated with zinc at the indicated molar ratios. For ZnuE, ZnuF, and IutA, 5 µM protein and 0–100 µM ZnCl_2_ were tested. For ZnuG, 10 µM protein and 0–200 µM ZnCl_2_ were tested. The thermal stability of proteins from 20°C to 95°C was monitored by the native fluorescence of the protein at 330 and 350 nm. (**A**) The ratio of 350 nm to 330 nm (top panel) and the first derivative of the ratio (bottom panel) are shown for ZnuE and ZnuG. The *x*-axis is truncated to visualize the results more clearly. (**B and C**) Only the first derivative of the ratio is shown with a temperature range from 30°C to 95°C. The *y*-axis shows the first derivative of the 350/330 nm ratio for each protein. Data for each protein are representative of replicate experiments. Inflection points (dotted lines) were calculated using PR.Stability Analysis software (version 1.0.3).

The growth results (Fig. 2 and 3) suggest that ZnuE, ZnuF, and ZnuG are involved in zinc metabolism, and that the conserved His residues in the HxD/HxH motif are critical for function. Furthermore, the predicted structures for the HxD/HxH motif exhibit similarities to the zinc-binding site in other well-characterized zinc-binding proteins (16–19). Thus, we proceeded to assess the potential for zinc binding using nano differential scanning fluorimetry (nanoDSF). The nanoDSF technique is a thermal shift assay that uses the native fluorescence of tryptophan side chains in each protein to monitor conformational changes; protein-ligand interactions stabilize or destabilize the protein to result in changes in thermal stability. We assayed the thermal stability of recombinant ZnuE, ZnuF, and ZnuG, both in the absence of zinc and with the addition of different concentrations of ZnCl_2_ (Fig. 4).

For ZnuE and ZnuG, we present the ratio of signal detected at 350 and 330 nm (Fig. 4A, top panels) and the first derivative of the ratio (bottom panels), which facilitates visualization of the inflection points and indicates the temperature where half of the protein is unfolded. Apo-ZnuE (dotted black line; Fig. 4A) shows two distinct inflection points at 54.07°C and 76.87°C; with the addition of Zn, only the higher temperature inflection point is observed, and protein stability is reduced (74.69°C at 20:1 [Zn]:[protein]). We note that at lower concentrations of Zn, ZnuE becomes more stable, but as the Zn concentration increases, ZnuE becomes less stable. These data suggest that the conformation of Apo-ZnuE is altered in the presence of Zn, and the concentration of Zn may influence how Zn binds to ZnuE. Apo-ZnuG also shows two distinct inflection points; one at 61.24°C, and the second beyond the temperature range assayed by the instrument (Fig. 4A). The lower inflection point is lost in the presence of Zn. While the higher temperature inflection point cannot be fully visualized, the ratios indicate that in the presence of zinc, ZnuG begins to unfold at a lower temperature, suggesting that ZnuG also binds Zn.

Apo-ZnuF, which contains two HxD/HxH domains, shows three distinct inflection points: 50.62°C, 67.01°C, and 78.96°C. Similar to ZnuE and ZnuG, the lowest-temperature inflection point is not observed in the presence of Zn, and consistent with ZnuE, lower ratios of Zn stabilize ZnuF, while higher ratios of Zn destabilize ZnuF (Fig. 4B). The inflection points at 67.01°C and 78.96°C both shift lower with the addition of Zn, which is consistent with the presence of two independent Zn binding sites with separate unfolding temperatures. We also tested IutA, which was previously shown to bind Zn by isothermal titration calorimetry (ITC) (9). Unlike the Znu proteins, IutA shows a reduction in the ratio with increasing temperature that results in the inflection points appearing as troughs instead of peaks; this is a characteristic of the protein and is not indicative of a difference in binding. Interestingly, apo-IutA, like the apo-Znu proteins, also shows two inflection points, and only one inflection point is observed with the addition of Zn. Consistent with previous ITC observations, the primary inflection point also shifts, which supports Zn binding by IutA (Fig. 4B).

Because we observed thermal shifts consistent with Zn binding, we also tested the ZnuE(3xA), ZnuG(3xA), and IutA(3xA) mutant derivatives. If the conserved His residues are critical for the observed binding, then Ala substitutions should abrogate the thermal shifts. ZnuE(3xA) shows reduced thermal stability in the absence of Zn (54.60°C) and further destabilization with the addition of Zn (down to 44.61°C) (Fig. 4C). Overall, reduced thermal stability may be expected due to the loss of His side chains with their ability to form stabilizing hydrogen bonds. However, the thermal shift of ZnuE(3xA) in response to Zn suggests that it retains the ability to bind Zn; the targeted residues may not be directly involved in Zn binding, or a second binding site may exist elsewhere in the protein. ZnuG(3xA) also shows overall reduced thermal stability compared to the wild-type protein; however, while the temperature onset of unfolding is unchanged, the inflection point changes from 58.70°C to 54.82°C. This may suggest Zn binding, but the results are unclear. We cloned and tested an IutA(3xA) mutant in which the three conserved His residues of the predicted substrate-binding site are substituted with Ala. Replacement of the His residues also greatly reduced the thermal stability of the protein (Fig. 4C). Like the ZnuE mutant, IutA(3xA) showed reduced thermal stability in response to Zn, which is consistent with our previous report of an additional, undefined Zn binding site (9).

Altogether, these data suggest that ZnuE, ZnuF, and ZnuG can bind Zn; however, the importance of the conserved motif remains unclear due to the binding observed in the His to Ala mutants. Based on the structural analysis of this motif, as well as the zinc transport studies using a mutated motif, it seems likely that the HxD/HxH motif is involved in Zn binding; however, additional studies will be required to establish definitive evidence for zinc binding to this motif.

### ZnuE is a membrane protein

**Fig 5:**
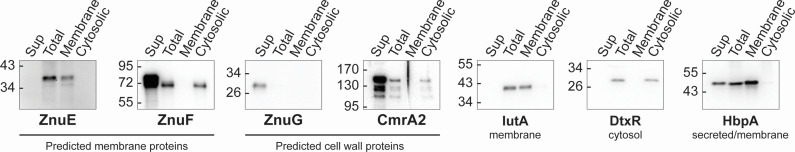
ZnuE is a membrane protein. Western blots of cell fractions from wild-type *C. diphtheriae* grown under zinc limitation. The culture supernatant (Sup), total clarified lysate (Total), and membrane and cytosolic fractions are shown. The total clarified lysate fraction contains both the membrane and cytosolic proteins. Control targets were probed to confirm successful enrichment of the membrane and cytosolic proteins: DtxR is a cytosolic protein; IutA is a membrane protein; and HbpA is a secreted/membrane protein.

Both the ZnuE and ZnuF proteins bind to zinc, have roles in zinc metabolism, and contain the novel HxD/HxH motif. The primary structure of these proteins predicts that they are both secreted and potentially associated with the cell membrane through a putative C-terminal transmembrane region, a structure similar to that present in the *C. diphtheriae* heme transport proteins (21, 22). To determine the localization of the ZnuE and ZnuF proteins, we used a previously described cell fractionation method to distinguish between membrane and cytosolic proteins (9). Consistent with its predicted localization, ZnuE was detected in the membrane fraction and total lysate, which includes membrane and cytosolic proteins (Fig. 5). Surprisingly, ZnuF was not detected in the membrane fraction but instead was predominantly observed in the culture supernatant. To assess the suitability of this fractionation technique, we showed that the localization of various control target proteins, IutA (membrane), DtxR (cytosol), and HbpA (secreted/membrane), was all observed in the expected cell fraction as reported previously (Fig. 5 and [9, 23]). We also examined the localization of ZnuG and CmrA2, which contain sortase signals and are predicted to be present in the cell wall. Using this membrane isolation technique, both ZnuG and CmrA2 were found primarily in the supernatant, similar to what was observed for ZnuF (Fig. 5). While this fractionation method can distinguish between membrane and cytosolic proteins, it is not intended for the characterization of cell wall proteins, since this localization technique results in the release of most of the cell wall proteins into the culture supernatant fraction. The results here indicate that ZnuE localizes to the cell membrane as predicted; however, ZnuF shows a localization pattern similar to what was observed with proteins ZnuG and CmrA that are predicted to reside in the cell wall.

### ZnuF and ZnuG are cell wall proteins

**Fig 6:**
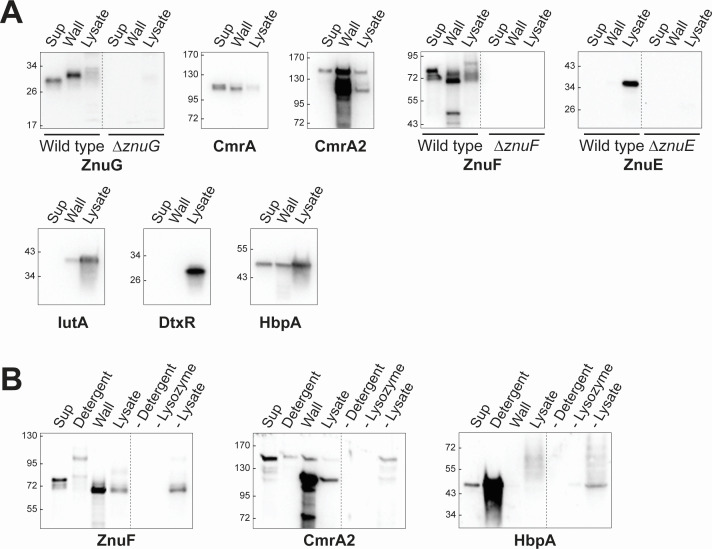
ZnuF shows cell wall localization. (**A**) Western blots of fractions from cell wall protein enrichment. Culture supernatants (Sup), proteins released from bacterial cells following incubation with lysozyme and mutanolysin (Wall), and remaining proteins (Lysate) are shown for the wild-type strain or indicated mutant used as a control to verify the antigens detected by the indicated antisera. Control targets were probed to confirm successful enrichment of cell wall proteins and separation of fractions. DtxR is a cytosolic protein; IutA is a membrane protein; and HbpA is a secreted/membrane protein. (**B**) Western blots of fractions collected from the sequential treatment of cells with detergent, followed by lysozyme and mutanolysin. Spheroplasts and cells were mechanically lysed and clarified by centrifugation to generate the lysate. For a mock treatment control processed in parallel, fractions were collected for a cell pellet subjected to the same sequential treatment using the same buffers, time, and temperatures; however, no detergent (−Detergent) or enzymes (−Lysozyme) were added for those steps. As with treated cells, the cells were lysed mechanically, and the clarified lysate was separated for blotting (−Lysate). Results for mock-treated samples are separated by the dotted line.

*C. diphtheriae* cell wall proteins were identified by treating cells with lysozyme and mutanolysin, followed by low-speed centrifugation, which separates the cell wall fraction from the intact cells and spheroplasts (Fig. 6A); culture supernatant was also examined to assess secreted proteins. Consistent with their predicted localization, ZnuG, CmrA, and CmrA2 were enriched in the cell wall fraction (Fig. 6A). Surprisingly, ZnuF was also present primarily in the cell wall fraction, which was unexpected since ZnuF lacks an identifiable sortase processing signal on its C-terminus. Varying levels of each of these cell wall proteins were also observed in the supernatant and lysate fractions. As expected, ZnuE was only detected in the lysate fraction, which is expected to contain membrane and cytosolic proteins with this treatment protocol. Control target proteins IutA, DtxR, and HbpA were used to confirm that the method demonstrated the expected fractionation (Fig. 6). IutA and DtxR are detected predominantly in the cell lysate fraction, consistent with their membrane and cytosolic localization, respectively (Fig. 6). Previous studies show that HbpA is detected in the membrane, supernatant, and on the cell surface, so its presence in each of the three fractions was expected (23).

The treatment of the cells with muramidase enzymes successfully released cell wall proteins, as observed in Fig. 6A. However, it is possible that secreted proteins, like HbpA, may associate with cell wall components, even though they are not specifically localized to the cell wall. We reasoned that the detection of ZnuF in the cell wall fraction could also be due to weak interactions and not targeted localization. Several proteins in gram-positive bacteria have been reported to be loosely associated with the cell wall through relatively weak ionic or hydrophobic interactions (24). Previously, we showed that HbpA is released from *C. diphtheriae* cells by detergent treatment, suggesting a weak non-covalent association with the cell envelope (21). If ZnuF is associated with the cell wall through weak or non-specific interactions, then it should also be released by detergent treatment. However, if ZnuF is tightly attached to the cell wall (i.e., covalently), enzymatic treatment with muramidase would be required for protein release. To test for either outcome, we modified our cell wall protein extraction protocol to include a detergent treatment step prior to enzymatic treatment (Fig. 6B). A mock-treated cell pellet was also incubated under the same buffer, temperature, and time conditions as the treated pellet, but without added detergent or enzyme. For ZnuF and CmrA2, most of the protein was found in the cell wall fraction with only weak signals detected in the detergent fraction, strongly suggesting that both proteins are tightly associated with the cell wall, consistent with covalent interactions as would be predicted for sortase-anchored proteins (Fig. 6B). HbpA was almost entirely removed from the cells with the detergent treatment, indicating a loose association with the bacterial surface, as reported previously (21). Together, the data suggest that ZnuF is likely covalently anchored to the cell wall, as indicated by its resistance to removal by detergent and the requirement for enzymatic treatment for it to be removed from the cell surface.

### ZnuF cell wall localization does not require known sortase enzymes

**Fig 7:**
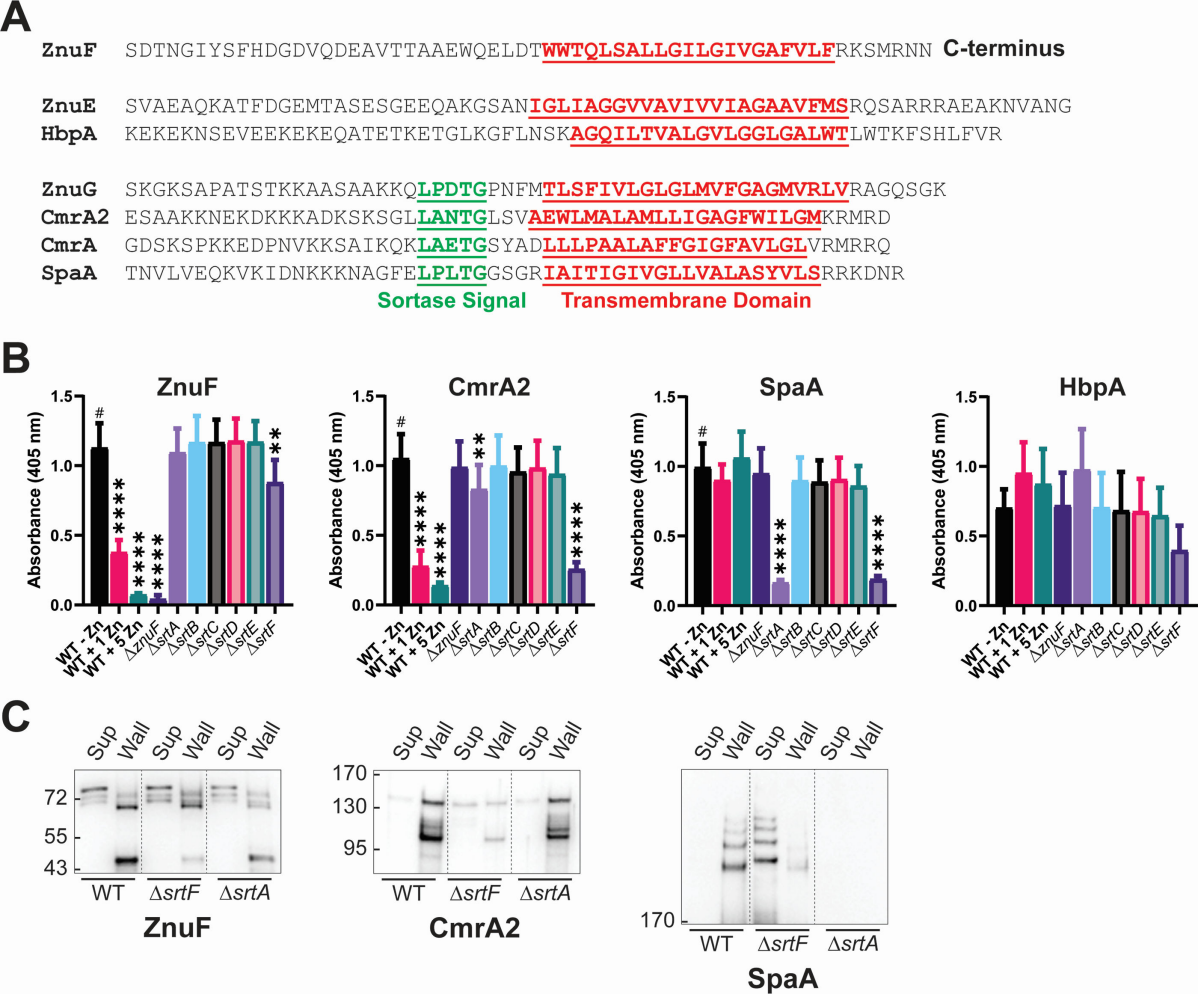
ZnuF localizes to the cell wall independent of *srtA-F*. (**A**) Amino acid sequence for the C-terminal region of the indicated proteins. The underlined red region indicates a sequence predicted to constitute a transmembrane domain. The underlined green region indicates the predicted sortase processing signal. (**B**) Whole cell ELISA data in which the indicated strains were used to coat ELISA plates; plates were probed with the indicated antibody and detected using an alkaline phosphatase-conjugated secondary antibody. The wild-type strain was grown either with no supplemented ZnCl_2_ (wild type [WT] −Zn), 1 µM (WT +1 Zn), or 5 µM (WT +5 Zn); all other strains were only grown without ZnCl_2_ supplementation. An ordinary one-way ANOVA Fisher’s LSD test was used to compare samples against results for the WT strain (#). **P < 0.01 and ****P < 0.0001; *n* ≥ 4. (**C**) Western blot of the culture supernatant (Sup) or cell wall proteins released by lysozyme/mutanolysin treatment (Wall) for indicated strains: wild type, Δ*srtF*, or Δ*srtA*. The high molecular weight band in the Sup lanes likely represents the mature protein for ZnuF and CmrA2.

The well-characterized *C. diphtheriae* strain NCTC 13129 is closely related to the 1737 strain used in this study. Strain NCTC 13129 (as well as strain 1737) is known to encode six sortase enzymes, and the roles for each sortase have been extensively characterized in the context of pilin biogenesis in strain NCTC 13129 (25–27). The housekeeping sortase, encoded by *srtF*, links proteins to cell wall precursors for integration into the cell wall and is required for efficient attachment of pili to the cell wall (25). The other sortase enzymes encoded by *srtA-E* are pilin-locus associated and involved in polymerization of their cognate pili. The known *C. diphtheriae* sortase enzymes predominantly recognize the amino acids LPXTG or LAXTG (27, 28) (Table 1). ZnuF lacks the C-terminal sortase processing site despite having the other requisite signals, including a signal sequence and a C-terminal transmembrane region followed by positively charged residues (Fig. 1B and 7A). To test if ZnuF localization requires any of the sortase enzymes encoded by *srtA-F*, we evaluated the impact of individual sortase gene deletions on protein localization using a whole cell ELISA method, which we previously used to assess cell surface exposure of proteins (9, 23). To confirm the ability of the method to detect changes in ZnuF protein levels, we tested varying zinc supplementation levels and an in-frame *znuF* deletion mutant. In wild-type *C. diphtheriae*, ZnuF was detected on the cell surface in zinc-limited medium, conditions in which ZnuF is optimally produced (Fig. 7B). As expected, reduced signal was detected for ZnuF from cells grown in media supplemented with 1 or 5 µM zinc (Fig. 7B) (10). The *znuF* deletion mutant (Δ*znuF*), used as a negative control, showed only baseline levels of signal when probed with ZnuF antibody.

**TABLE 1:** Putative and confirmed *C. diphtheriae* cell wall proteins^*b*^

Gene locus	Protein	Description	Sortase signal^*a*^	Reference
*dip0235*	SpaD	SpaDEF major subunit	L**P**MTG	(26, 29)
*dip0237*	SpaE	SpaDEF basal subunit	L**A**LTG	(29)
*dip0238*	SpaF	SpaDEF tip protein	L**P**KTG	(29)
*dip0278*		Putative surface-anchored membrane protein	L**A**RTG	
*dip0443*	ZnuG	Surface-anchored membrane protein	L**P**DTG	
*dip1724*	CmrA2	Surface-anchored membrane protein	L**A**NTG	
*dip2010*	SpaC	SpaABC tip protein	L**P**LTG	(26, 29)
*dip2011*	SpaB	SpaABC basal subunit	L**A**FTG	(26, 29, 30)
*dip2013*	SpaA	SpaABC major subunit	L**P**LTG	(26, 29)
*dip2062*		Surface-anchored protein	L**A**ATG	
*dip2066*		Surface-anchored fimbrial associated protein	L**P**KTG	
*dip2093*		Sdr-family related adhesin	L**A**ATG	(31)
*dip2223*	SpaI	SpaGHI subunit	L**G**NTG	(26)
*dip2226*	SpaH	SpaGHI major subunit	L**P**LTG	(26)
*dip2227*	SpaG	SpaGHI tip protein	L**P**LTG	(26)
*dip2325*	CmrA	Surface-anchored protein	L**A**ETG	
*dip2370*		Putative secreted protein (DUF6049)	L**L**STG	

^*a*^ Bold letters indicate the semi-conserved amino acid residue found in the second position of the pentapeptide sortase signal.

^*b*^ Empty cells indicate the absence of prior studies or annotation for the protein product.

Of the six sortase mutants tested, only the deletion of *srtF* resulted in a decrease in ZnuF signal. However, the Δ*srtF* strain shows reduced binding to ELISA plates that is reflected by the reduced detection of HbpA, which localizes to the surface by a sortase-independent mechanism (Fig. 7B). The reduced binding of the Δ*srtF* strain to the ELISA plates likely explains the decreased signal observed for ZnuF in the Δ*srtF* strain. For CmrA2, we also found that zinc supplementation during cell growth impacted the amount of protein detected on the cell surface, consistent with the known repression of CmrA2 by zinc. CmrA2 detection was dramatically reduced on the cell surface in the Δ*srtF* mutant, suggesting that SrtF is likely responsible for the majority of CmrA2 anchoring to the cell wall and is consistent with SrtF functioning as the housekeeping sortase responsible for anchoring proteins to the cell wall. In contrast, the Δ*srtA* mutant showed a modest reduction in the signal for CmrA2, and it is unclear if *srtA* impacts CmrA2 localization. As an additional control for these studies, we examined the localization of the SpaA pilus protein, a well-characterized sortase-anchored protein that is polymerized by *srtA* and then anchored to the cell wall by *srtF* (20). Consistent with previous studies, SpaA was poorly detected on the cell surface in both the *srtF* and *srtA* mutants (Fig. 7B) (25).

To further evaluate the potential role of *srtF* on ZnuF localization and reconcile the results observed with *srtA* and CmrA2, we performed cell wall preparations with the *srtF* and *srtA* mutants (Fig. 7C). Culture supernatant and cell wall fractions from wild-type *C. diphtheriae* and isogenic *srtF* and *srtA* mutants were probed for ZnuF, CmrA2, and SpaA. ZnuF levels, in general, showed a similar signal across the different strains, with some variations in the breakdown products in the different cell fractions. Sharply reduced levels of the CmrA2 protein were detected in the Δ*srtF* cell wall fraction, consistent with the results in Fig. 7B showing a dependence on SrtF for cell wall anchoring. CmrA2 levels in the *srtA* mutant did not appear to be different from the wild-type strain, suggesting that SrtA does not have a significant role in CmrA2 anchoring to the cell wall (Fig. 7C). Polymerized SpaA levels were significantly reduced in the cell wall fraction in both the *srtF* and *srtA* mutants as expected (Fig. 7C). In the absence of *srtF*, polymerized SpaA is not efficiently integrated into the cell wall, resulting in release into the culture supernatant; and without *srtA*, SpaA does not polymerize (25, 26). Together, these data suggest that ZnuF does not traffic to the cell wall in a manner dependent on any of the six known *C. diphtheriae* sortase enzymes. Additional studies are needed to better understand how ZnuF is localized to the cell wall.

## DISCUSSION

Bacterial pathogens use a wide variety of tools to scavenge for essential nutrients, including metals such as zinc. Known mechanisms for zinc import by gram-positive bacteria, such as *C. diphtheriae,* typically fall under one of three general categories: ABC transporters, metallophores, and ZIP/ZupT family proteins (4). In our previous study, we found that *C. diphtheriae* encodes several ABC transporters involved in zinc uptake and a putative metallophore biosynthetic locus with associated transporters necessary to import complexed zinc (8). The three ABC transporters with substrate-binding components predicted to bind zinc include Iut, Mnt, and Znu; each of these systems has unique characteristics that differ from the canonical zinc transporters described in other bacteria. The *iutABCD*/*E* gene cluster encodes two distinct and independently expressed substrate-binding proteins, IutA and IutE, in addition to permease and ATPase components (8, 9). The *mntABCD* locus encodes an Mn/MntR-regulated transporter, which was previously shown to be involved in Mn transport (11) and was later demonstrated to also function in zinc uptake (8).

In this study, we further characterized the *znu* gene cluster, which comprises the two zinc-regulated operons *znuAECB* and *znuF-445*. We found that the novel membrane protein encoded by *znuE* appears to be critical for zinc transport, since it exhibited significantly reduced growth in zinc-limited medium, while the substrate-binding protein encoded by *znuA* is less important in zinc uptake based on growth studies under low zinc conditions (Fig. 3A). A reason for the weak phenotype for the *znuA* mutant may be due to other substrate-binding proteins compensating for the loss of the ZnuA function. Furthermore, it is unclear what role the proteins encoded in the *znuF-445* operon have on the function of the *znuAECB*-encoded transporter, as the strain deleted of *znuF-dip0445* (Δ5 Δ*znuF-445*) grew to a similar peak density as the isogenic parent strain, but showed a longer lag phase, which may indicate an impairment in zinc transport (Fig. 2B and C). Surprisingly, the reduced growth observed in the Δ5 Δ*znuAECB* strain is restored to WT peak density when the Δ*znuF-445* locus is also deleted, generating strain Δ5 Δ*znuA-445* (Fig. 2A through C). This unusual phenotype is dependent upon the function of the proteins encoded by *znuF* and *znuG* (Fig. 2D and E), which are cell wall-anchored zinc-binding proteins that may serve to sequester zinc on the cell surface in the absence of the ZnuAECB zinc transporter. This binding to zinc on the surface of the bacteria by ZnuF and ZnuG may account for the severe growth reduction in zinc-limited medium that is observed when these proteins are over-expressed from plasmids (Fig. 2E). A possible function for ZnuF and ZnuG may be to facilitate the transport of zinc through the ZnuAECB transporter or possibly through one of the other high-affinity zinc transport systems (8). It is also possible that ZnuF and ZnuG may function as storage proteins for zinc, in which subsequent transport of zinc may occur during growth in zinc-limited environments. Further studies are needed to understand the precise roles of ZnuF, ZnuG, and the related surface-exposed proteins CmrA/CmrA2; however, we maintain that the HxD/HxH motif is critical for the function of these proteins as the conserved His residues are important for both the transport function of ZnuE and the growth-limiting function observed for ZnuG (Fig. 2D, E, 3C, and D). While we were able to demonstrate a requirement for the conserved His residues in growth assays, we were unable to conclusively show a role for the residues in *in vitro* binding to zinc. Despite the importance of the HxD/HxH motif in ZnuE and ZnuG, which encode only a single domain, the significance of multiple motifs found in ZnuF, CmrA, and CmrA2 is unclear. It is possible they may have a similar function to the multiple zinc binding domains found in the polyhistidine triad proteins (Pht) in *Streptococci* (32).

The ZnuF, ZnuG, and CmrA proteins, while distinct in amino acid sequence, share characteristics with the Pht proteins found in *Streptococci* (32–36). Extensive studies have been conducted to evaluate the function of Pht proteins and their potential as vaccine candidates for *S. pneumoniae* (37). These proteins have been shown to have multiple functions, including zinc binding through a conserved His-x-x-His-x-His motif present in multiple copies (up to six) in each Pht protein (35). Pht proteins are found on the bacterial cell surface and are proposed to deliver zinc ions to the substrate-binding protein, AdcAII, for transport through the membrane (33). *S. pneumoniae* PhtD is also found in the cell wall and is present in high abundance in the culture supernatant (35, 38). The family of *S. pneumoniae* Pht proteins is composed of surface-exposed antigens that, like ZnuF in *C. diphtheriae*, do not appear to require sortase enzymes for their localization (32, 36, 38). However, amino acids R26, H27, and Q28 in PhtD and Q27, H28, and R29 in PhtE are important for the surface localization of each respective protein, but the residues are not conserved among the other Pht proteins (38). While each protein may use different mechanisms for localization, how the Pht proteins localize to the cell wall or other compartments in the cell envelope is not well understood. It is important to note that while the cell fractionation methods used to assess the Pht proteins in *S. pneumoniae* are similar to those we use in this study, it is unclear from other reports whether all of the Pht proteins are covalently linked to the cell wall. To our knowledge, sequential treatment of cells with detergent followed by muramidase was not tested for the Pht proteins. However, Plumptre and colleagues (38) showed that Pht proteins released from wild-type *S. pneumoniae* cells do not bind to the bacterial cell surface of a *pht* deletion mutant, which would suggest that the proteins do not associate with the cell surface in a manner similar to the secreted HbpA protein in *C. diphtheriae* (21).

In this study, we showed that ZnuF, ZnuG, and CmrA/CmrA2 are localized to the cell wall and present at varying amounts in the culture supernatant. While ZnuG, CmrA, and CmrA2 have identifiable sortase processing sequences (Fig. 7A), ZnuF lacks sequences resembling any known sortase signal in its C-terminal region. The mechanism by which proteins traffic to the cell wall is well studied in *C. diphtheriae* in the context of the three distinct pili encoded in *C. diphtheriae* NCTC13129 (26, 29). Previously described *C. diphtheriae* cell wall proteins require SrtF for integration into the cell wall, and the other sortase enzymes associated with the pilin loci are required for polymerization of the respective pilus components. In addition to the requirement for a sortase enzyme, *C. diphtheriae* cell wall proteins require an N-terminal signal sequence and a C-terminal sortase processing site followed by a transmembrane region for localization. The sortase processing site, specifically the Thr residue, is critical for the formation of the amide bond. For pilin proteins that are polymerized to one another, an additional pilin motif (WxxxVxVYPK) within the protein is also required for protein-protein attachment (26, 29). We were unable to identify a sortase signal in the C-terminal sequence of ZnuF, even when searching for sortase recognition sequences found in other organisms that use different sortase enzymes (39). Consistent with the absence of a known sortase signal, the deletion of sortase genes did not impact the detection of ZnuF in the cell wall, as it did for CmrA2 or SpaA, which both have a clear requirement for *srtF,* as well as *srtA* for SpaA. The absence of a sortase signal and no requirement for any of the known sortase genes suggest that ZnuF cell wall localization uses a novel mechanism in *C. diphtheriae*. The identification of the signals and protein(s) required for ZnuF localization may expand our understanding of cell wall proteins in *Corynebacteria* and potentially other related organisms.

## MATERIALS AND METHODS

### Bacterial strains, plasmids, and growth media

Bacterial strains and plasmids used in this study are listed in Table 2. *C. diphtheriae* strains were routinely grown in heart infusion broth (HIB) (BD Difco) with 0.2% (vol/vol) Tween 80 at 37°C (HIBTW) or on heart infusion agar (1.5% [wt/vol]) at 30°C or 37°C. Strains were stored at −80°C in HIB with 20% (vol/vol) glycerol. mPGT medium containing 0.5% (wt/vol) Casamino Acids treated with Chelex 100 and supplemented with 1 µM FeCl_3_ was used for general growth. The composition of mPGT is described in reference 8. The zinc levels in mPGT have not been measured and may exhibit some variability due to the nature of Chelex treatment of Casamino Acids; however, wild-type *C. diphtheriae* strain 1737 shows improved growth in mPGT medium with the addition of zinc (1 µM ZnCl_2_) shown in reference 8. Antibiotics were used at 25 µg/mL for kanamycin and 10 µg/mL for nalidixic acid.

**TABLE 2:** Strains and plasmids used in this study

Strain or plasmid	Description or use	Reference or source
*C. diphtheriae* strains		
1737	Wild type, Gravis biotype, Tox^+^	(40)
Δ5 Δ*znuAECB*	Δ*iutABCD/E* Δ*znuAECB* Δ*nikABCD1* Δ*nikABCD2* ΔmntABCD Δ*sidAB;* in the source reference, this strain was described as Δ6 and has been renamed herein for clarity.	(8)
Δ5 *znu*^+^	Δ*iutABCD/E* Δ*nikABCD1* Δ*nikABCD2* ΔmntABCD Δ*sidAB*	(8)
Δ5 Δ*znuF-445*	Δ*znuF-dip445* Δ*iutABCD/E* Δ*nikABCD1* Δ*nikABCD2* ΔmntABCD Δ*sidAB*	This study
Δ5 Δ*znuA-445*	Δ*iutABCD/E* Δ*znuAECBFG-dip444-dip0445* Δ*nikABCD1* Δ*nikABCD2* ΔmntABCD Δ*sidAB*	This study
Δ5 Δ*znuA*	Δ*znuA* Δ*iutABCD/E* Δ*nikABCD1* Δ*nikABCD2* ΔmntABCD Δ*sidAB*	This study
Δ5 Δ*znuE*	Δ*znuE* Δ*iutABCD/E* Δ*nikABCD1* Δ*nikABCD2* ΔmntABCD Δ*sidAB*	This study
Δ5 Δ*znuF*	Δ*znuF* Δ*iutABCD/E* Δ*nikABCD1* Δ*nikABCD2* ΔmntABCD Δ*sidAB*	This study
Δ5 Δ*znuG*	Δ*znuG* Δ*iutABCD/E* Δ*nikABCD1* Δ*nikABCD2* ΔmntABCD Δ*sidAB*	This study
Δ*srtA*	Δ*srtA* (*dip2012*)	This study
Δ*srtB*	Δ*srtB* (*dip0233*)	This study
Δ*srtC*	Δ*srtC* (*dip0236*)	This study
Δ*srtD*	Δ*srtD* (*dip2224*)	This study
Δ*srtE*	Δ*srtE* (*dip2225*)	This study
Δ*srtF*	Δ*srtF* (*dip2272*)	This study
*E. coli* strains		
S17-1 λpir	Mating strain	(41)
NEB 5-alpha	Cloning strain	New England Biolabs, Inc.
BL21(DE3)	Protein expression strain	New England Biolabs, Inc.
Plasmids		
pKΔ*znuF-445*	Suicide vector for the deletion of the *znuFG-dip0444-45* (*dip0442-45*) operon	This study
pKΔ*znuA-445*	Suicide vector for the deletion of the *znuAECB* and *znuFG-dip0444-45* (*dip0438-45*) operons	This study
pKΔ*znuA*	Suicide vector for the in-frame deletion of *znuA* (*dip0438*)	This study
pKΔ*znuE*	Suicide vector for the in-frame deletion of *znuE* (*dip0439*)	This study
pKΔ*znuF*	Suicide vector for the in-frame deletion of *znuF* (*dip0442*)	This study
pKΔ*znuG*	Suicide vector for the in-frame deletion of *znuG* (*dip0443*)	This study
pKΔ*srtA*	Suicide vector for the in-frame deletion of *srtA* (*dip2012*)	This study
pKΔ*srtB*	Suicide vector for the in-frame deletion of *srtB* (*dip0233*)	This study
pKΔ*srtC*	Suicide vector for the in-frame deletion of *srtC* (*dip0236*)	This study
pKΔ*srtD*	Suicide vector for the in-frame deletion of *srtD* (*dip2224*)	This study
pKΔ*srtE*	Suicide vector for the in-frame deletion of *srtE* (*dip2225*)	This study
pKΔ*srtF*	Suicide vector for the in-frame deletion *srtF* (*dip2272*)	This study
pKN2.6Z	*C. diphtheriae* shuttle vector; kan^R^	(42)
pKN-*znuF-445*	*znuF*(*dip0442*)*-dip0445* cloned into pKN2.6Z with native promoter region	This study
pKN-*znuF*	*znuF* (*dip0442*) cloned into pKN2.6Z with pO5 promoter	This study
pKN-*znuG*	*znuG* (*dip0443*) cloned into pKN2.6Z with pO5 promoter	This study
pKN-*znuG*(3xA)	*znuG* (*dip0443*) with His to Ala mutations in the conserved residues cloned into pKN2.6Z with pO5 promoter	GenScript
pKN-*dip0444*	*dip0444* cloned into pKN2.6Z with pO5 promoter	This study
pKN-*dip0445*	*dip0445* cloned into pKN2.6Z with pO5 promoter	This study
pKN-*znuE*	*znuE* (*dip0439*) cloned into pKN2.6Z with pO5 promoter	This study
pKN-*znuE*(3xA)	*znuE* (*dip0439*) with His to Ala mutations in the conserved residues cloned into pKN2.6Z with pO5 promoter	This study
pSII-znuE	*znuE* (*dip0439*) cloned into pET24a with an N-terminal Strep II tag	This study
pSII-znuE(3xA)	*znuE* (*dip0439*) with His to Ala mutations in the conserved residues cloned into pET24a with an N-terminal Strep II tag	This study
pSII-znuF	*znuF* (*dip0442*) cloned into pET24a with an N-terminal Strep II tag	This study
pSII-znuG	*znuG* (*dip0443*) cloned into pET24a with an N-terminal Strep II tag	This study
pSII-znuG(3xA)	*znuG* (*dip0443*) with His to Ala mutations in the conserved residues cloned into pET24a with an N-terminal Strep II tag	This study
pSII-iutA	*iutA* (*dip0169*) cloned into pET24a with an N-terminal Strep II tag	This study
pSII-iutA(3xA)	*iutA* (*dip0169*) with His to Ala mutations in the conserved residues cloned into pET24a with an N-terminal Strep II tag	This study
pET1725	*cmrA2* (*dip1724*) lacking signal sequence cloned into pET30a	This study
pCmrAHIS	*cmrA (dip2325*) lacking signal sequence into pET30a	This study

*E. coli* strains used for cloning and protein expression were routinely grown in Luria Broth (LB) (BD Difco) or on LB agar (1.5% [wt/vol]) at 37°C. Strains were stored at −80°C LB with 20% (vol/vol) glycerol. Strains for protein expression were grown in Overnight Expression Autoinduction Media (Novagen) at 30°C. Kanamycin was used at 50 µg/mL where appropriate.

### Cloning and *C. diphtheriae* mutant generation

All DNA sequences used in this study were derived from *C. diphtheriae* strain 1737 and amplified by PCR from *C. diphtheriae* strain 1737 genomic DNA or synthesized based on the reported sequence of *C. diphtheriae* NCTC 13129 (43) (GenScript).

Target gene deletion was performed by allelic exchange using *E. coli* strain S17-1 λpir for conjugation as described in reference 26 and suicide plasmids listed in Table 2. Suicide plasmid constructs were designed by fusing approximately 500 bp each of flanking genomic regions of the target genes, resulting in <18 total amino acids from the first and last gene of the target locus or individual target gene (44). Isolates were tested for kanamycin sensitivity and sucrose resistance (HIA with 10% [wt/vol] sucrose). Target gene deletion was verified by PCR across the gene locus using primers external to the sequence in the suicide plasmid.

Complementation plasmids were created in the pKN2.6Z plasmid backbone (42). For pKN-*znuF-445*, the genomic locus and native promoter were cloned into pKN2.6Z. For plasmids in which only one gene was introduced, the pO5 promoter of the *mntA* promoter region (8, 20) was fused to the start codons of cloned genes to ensure expression. Site-directed mutagenesis was performed using primers designed with the intended nucleotide sequence for PCR amplification or gene synthesis (GenScript).

Protein expression plasmids were created using the pET24a vector in which the N-terminal region was replaced with a Strep II tag. Native gene sequences encoding the signal peptides and C-terminal transmembrane regions were removed. Plasmids were cloned using *E. coli* NEB 5-α (New England Biolabs, Inc.) and transformed into *E. coli* BL21(DE3) competent *E. coli* (New England Biolabs, Inc.) for expression and protein purification. pET30a vector was used for cloning and expression of His-tagged CmrA and CmrA2.

### Growth assays

Growth assays were performed as described previously (8). Strains were grown on HIA with antibiotics as appropriate from −80°C freezer stocks. Colonies were used to inoculate 1 mL of mPGT cultures. mPGT cultures were grown overnight with shaking (16–20 h, 37°C); overnight cultures were diluted with 1 mL of mPGT and incubated for 4–6 h at 37°C with shaking. Culture density (OD_600_) was measured, and cultures were used to inoculate 1 mL of mPGT in 2 mL volume deep well plates at a target OD_600_ of 0.03. Suspensions were mixed by pipetting and aliquoted into a 96-well microtiter plate (200 µL per well). Microtiter plates were sealed using gas-permeable sealing film, and the OD_600_ was monitored over 23 h, with data recorded in 5-min increments using a Varioskan LUX microplate reader with shaking and temperature control at 37°C. Baseline subtraction was performed for all samples to remove background absorbance introduced by the gas-permeable sealing film.

### Protein purification and antibody generation

Recombinant proteins were purified from *E. coli* cultures induced using Overnight Expression Autoinduction Media (Novagen). Cell pellets were lysed using 0.1 mm silica beads, and the crude lysates were clarified by centrifugation (>7,000 × *g*, 15 min, 4°C). Proteins were affinity purified using Streptactin XT 4flow resin (IBA Lifesciences) or HisTALON Metal Affinity Resin (Takara Bio USA, Inc.) following the manufacturer’s recommendations for gravity flow. Resins were washed using recommended buffers and eluted following the manufacturer’s protocols. Elution fractions containing the target protein were dialyzed against PBS and used to immunize guinea pigs for the production of antisera (Cocalico Biologicals, Inc.).

### Thermal shift assay by nanoDSF

Recombinant proteins were purified using Streptactin XT 4flow resin (IBA Lifesciences) following the manufacturer’s recommendations with modifications. EDTA was added to a final concentration of 10 mM in lysis and wash buffer (Buffer W). Buffer W with 1 mM EDTA was used for the final wash prior to elution with Buffer BXT. Proteins were buffer exchanged into 55.55 mM HEPES, pH 7.3, and 166.66 mM NaCl using Pierce PES Protein Concentrator with a 10,000 molecular weight cutoff. Protein concentrations were measured using the Agilent 2100 BioAnalyzer System and Agilent Protein 230 kit per the manufacturer’s instructions. Proteins were diluted to 5.55 µM based on predicted molecular weight (or 11.11 µM for ZnuG). ZnCl_2_ stock solutions (10× concentration) were prepared in water and diluted into the binding experiment to the final indicated concentrations (1 µL ZnCl_2_ solution to 9 µL protein/buffer) to result in 5 µM of protein (or 10 µM for ZnuG) with 50 mM HEPES, pH 7.3, and 150 mM NaCl.

Samples were analyzed using a Prometheus NT.48 (NanoTemper) in High Sensitivity Capillaries with a temperature ramp rate of 0.5°C/min from 20°C to 95°C. Fluorescence intensity was measured at 330 and 350 nm. The data presented are the ratio of fluorescence or the first derivative of the 350/330 nm ratio; a representative experiment is shown for each protein.

### Bioinformatics

Signal peptides were predicted using SignalP 6.0 (45). Transmembrane regions were predicted using TMHMM 2.0 (46, 47). ClustalW (48) and MEME (15) were used for protein sequence alignment and to generate a consensus motif. Predicted protein structures were obtained from the AlphaFold Protein Structure Database (16, 17). PyMOL 3.1.1. (Schrödinger, LLC) was used to visualize protein structures and produce images.

### SDS-PAGE and Western blotting

Protein samples were mixed with Laemmli sample buffer to a final concentration of 1× or 2× and heated to 98°C for 10 min. Sample loading volumes were generally normalized against the original culture OD_600_ prior to any further processing. Culture supernatant volumes used for SDS-PAGE were normalized against cell culture OD_600_ prior to cell harvest. Proteins were separated on 4%–15% SDS-PAGE (Bio-Rad Laboratories). Proteins were detected by Coomassie stain or Western blotting. Western blots were blocked in TBST with 5% blotting grade blocker (Bio-Rad Laboratories, Inc.). Antisera were used at indicated dilutions: αZnuE (1:2,500), αZnuF (1:2,500), αZnuG (1:2,500), αCmrA (1:5,000), αCmrA2 (1:5,000), αDtxR (1:5,000), αIutA (1:2,500), αHbpA (1:10,000), and αSpaA (1:5,000). Goat anti-guinea pig and goat anti-rabbit horseradish peroxidase-conjugated antibodies were used at dilutions of 1:50,000 and 1:5,000, respectively.

### Cell fractionations and protein localization studies

For studies involving protein localization, bacterial cultures were initiated as follows. Strains were grown on HIA from −80°C freezer stocks. Colonies were used to inoculate 1 mL of mPGT cultures. mPGT cultures were grown overnight with shaking (16–20 h, 37°C, and 250 rpm); overnight cultures were diluted with an additional 1 mL of mPGT and incubated for 4–6 h at 37°C with shaking. Culture density (OD_600_) was measured, and cultures were used to inoculate the appropriate volume of mPGT at a target OD_600_ of 0.03. Cultures were grown overnight and further processed the next day.

#### Subcellular fractionation

The bacterial cytosolic membrane and cytosolic fractions were separated as described previously with modifications (9). Cells were harvested by centrifugation (10 min, 4,000 × *g*, and 4°C) and resuspended in PBS. Culture supernatant was collected and centrifuged for 10 min at 21,000 × *g* to remove bacterial cells. Resuspended cell pellets were lysed using 0.1 mm silica beads (Lysing Matrix B, MP Biomedicals); crude lysate was centrifuged (15 min, 21,000 *× g*, and 4°C) to remove cell debris. A fraction of the clarified lysate was collected for SDS-PAGE, and the remainder was subjected to ultracentrifugation at 135,000 × *g* for 90 min at 4°C. Following ultracentrifugation, the supernatant containing cytosolic proteins and the pellet containing membrane proteins were further processed.

For the cytosolic fraction, the supernatant fraction was collected, and the ultracentrifugation was repeated; the resultant supernatant containing cytosolic proteins was collected for SDS-PAGE. For the membrane fraction, the membrane pellet was resuspended in PBS, and the ultracentrifugation was repeated; the supernatant was discarded, and the pellet was resuspended using 2× Laemmli sample buffer. Fractions were separated by SDS-PAGE.

#### Cell wall fractionation

Cell wall fractionation was performed as described by Swaminathan and colleagues with modifications (25). Cells were harvested by centrifugation (10 min, 4,000 *× g*, and 4°C). Culture supernatant was collected and centrifuged for 10 min at 21,000 *× g* to remove bacterial cells; supernatant samples were stored at −20°C until use. The cell pellet was resuspended in Buffer SMM (0.5 M sucrose, 10 mM MgCl_2_, and 10 mM maleate, pH 6.8), and cells were pelleted again to remove residual culture media (10 min, 4,000 *× g*, and 4°C). Washed cell pellets were resuspended in Buffer SMM with 100 U mutanolysin from *Streptomyces globisporus* ATCC 21553 (Millipore Sigma) and 150 kU rLysozyme (Millipore Sigma). The cell suspensions were incubated at 37°C with occasional agitation for at least 3 h. Spheroplasts and unlysed cells were removed by centrifugation (5 min, 4,000 × *g*, and 4°C); the supernatant containing released cell wall proteins was clarified by centrifugation at 21,000 × *g* for 15 min at room temperature to remove debris. The soluble fraction was collected for SDS-PAGE; pelleted material was discarded. The spheroplast and unlysed cells fraction was boiled in 2× Laemmli sample buffer for 10 min; lysates were clarified by centrifugation at 21,000 *× g* for 10 min at room temperature, and the soluble fraction was collected for SDS-PAGE.

#### Sequential detergent extraction and cell wall fractionation

Cells were harvested by centrifugation (10 min, 4,000 *× g*, and 4°C). Culture supernatant was collected and centrifuged for 10 min at 21,000 *× g* to remove bacterial cells; supernatant samples were stored at −20°C until use. The cell pellet was washed once with Buffer SMM and pelleted again to remove residual culture media (10 min, 4,000 × *g*, and 4°C). The cell pellet was resuspended in Buffer SMM containing 1% Triton X-100; the cell suspension was incubated at room temperature with mixing for 30 min. Cells were harvested by centrifugation (10 min, 4,000 × *g*, and 4°C); the supernatant containing detergent-solubilized proteins was clarified by centrifugation (15 min, 21,000 × *g*, and room temperature) and collected for SDS-PAGE. The cells were resuspended in Buffer SMM with 100 U mutanolysin and 150 kU rLysozyme, as above. Cell suspensions were incubated at 37°C for at least 3 h. Spheroplasts and unlysed cells were removed by centrifugation (5 min, 4,000 × *g*, and 4°C); the supernatant containing released cell wall proteins was clarified by centrifugation at 21,000 *× g* for 15 min at room temperature to remove debris. The soluble fraction was collected for SDS-PAGE, and pelleted material was discarded. The spheroplast and unlysed cells were lysed using 0.1 mm silica beads; lysates were clarified by centrifugation at 21,000 × *g* for 10 min at room temperature, and the soluble fraction was collected for SDS-PAGE. An equivalent cell pellet was processed concurrently with the same buffers, incubation periods, and centrifugation steps with fractions collected in parallel but no added detergent and enzyme as a negative treatment control; as with the treated cells, the cells following the final incubation were lysed mechanically, and the lysates were clarified by centrifugation under the same conditions.

#### Whole-cell ELISA

Whole-cell ELISAs were performed as described previously with modifications (9). The optical density (OD_600_) of bacterial cultures grown as described above was measured. A culture volume equivalent to an OD_600_ of 1 in 1 mL was pelleted for 5 min at 4,000 × *g* at room temperature. The cell pellets were resuspended in 1 mL sterile PBS, and 100 µL aliquots of the suspensions were added to a 96-well high-binding EIA/RIA plate (Corning Incorporated); plates were incubated for 1 h at 37°C. Unbound cells were removed, and the plates were blocked using PBST with 5% blotting grade blocker (Bio-Rad Laboratories, Inc.) (PBST-BB) for 30 min at 37°C. Primary antibodies were diluted in PBST-BB and incubated with bound cells for 1 h at 37°C at the following dilutions: αZnuF (1:2,500), αCmrA2 (1:5,000), αHbpA (1:5,000), and αSpaA (1:5,000). Alkaline phosphatase-linked secondary antibody was diluted 1:1,000 in PBST-BB and incubated with samples for 1 h at 37°C. *p*-nitrophenyl phosphate was added to wells to detect signal at 405 nm following incubation. ELISA data were read using a Varioskan LUX microplate reader in which plates were incubated at 37°C and data collected every 2 min over 30 min. The time point at which the wild type exhibited *A*_405_ closest to 1 for each antigen is presented for all strains.

### Statistical analyses

GraphPad Prism version 10.2.3 was used for the analysis of data. Specific tests and *P* values are noted in the figure legends.

## SUPPLEMENTAL MATERIAL

Figure S1 jb.00239-25-s0001.pdf Amino acid alignment.

ASM does not own the copyrights to Supplemental Material that may be linked to, or accessed through, an article. The authors have granted ASM a non-exclusive, world-wide license to publish the Supplemental Material files. Please contact the corresponding author directly for reuse.

## References

[1] Andreini C, Banci L, Bertini I, Rosato A. 2006. Zinc through the three domains of life. J Proteome Res 5:3173–3178. doi:10.1021/pr0603699 17081069

[2] Kozlyuk N, Monteith AJ, Garcia V, Damo SM, Skaar EP, Chazin WJ. 2019. S100 proteins in the innate immune response to pathogens. Methods Mol Biol 1929:275–290. doi:10.1007/978-1-4939-9030-6_18 30710280 PMC6475579

[3] Kehl-Fie TE, Skaar EP. 2010. Nutritional immunity beyond iron: a role for manganese and zinc. Curr Opin Chem Biol 14:218–224. doi:10.1016/j.cbpa.2009.11.008 20015678 PMC2847644

[4] Murdoch CC, Skaar EP. 2022. Nutritional immunity: the battle for nutrient metals at the host-pathogen interface. Nat Rev Microbiol 20:657–670. doi:10.1038/s41579-022-00745-6 35641670 PMC9153222

[5] Plumptre CD, Eijkelkamp BA, Morey JR, Behr F, Couñago RM, Ogunniyi AD, Kobe B, O’Mara ML, Paton JC, McDevitt CA. 2014. AdcA and AdcAII employ distinct zinc acquisition mechanisms and contribute additively to zinc homeostasis in Streptococcus pneumoniae. Mol Microbiol 91:834–851. doi:10.1111/mmi.12504 24428621

[6] Grim KP, San Francisco B, Radin JN, Brazel EB, Kelliher JL, Párraga Solórzano PK, Kim PC, McDevitt CA, Kehl-Fie TE. 2017. The metallophore staphylopine enables Staphylococcus aureus to compete with the host for zinc and overcome nutritional immunity. MBio 8:e01281-17. doi:10.1128/mBio.01281-17 29089427 PMC5666155

[7] Zackular JP, Knippel RJ, Lopez CA, Beavers WN, Maxwell CN, Chazin WJ, Skaar EP. 2020. ZupT facilitates Clostridioides difficile resistance to host-mediated nutritional immunity. mSphere 5:e00061-20. doi:10.1128/mSphere.00061-20 32161145 PMC7067591

[8] Peng ED, Lyman LR, Schmitt MP. 2024. Identification and characterization of zinc importers in Corynebacterium diphtheriae. J Bacteriol 206:e0012424. doi:10.1128/jb.00124-24 38809016 PMC11332173

[9] Peng ED, Oram DM, Battistel MD, Lyman LR, Freedberg DI, Schmitt MP. 2018. Iron and zinc regulate expression of a putative ABC metal transporter in Corynebacterium diphtheriae. J Bacteriol 200:e00051-18. doi:10.1128/JB.00051-18 29507090 PMC5915790

[10] Peng ED, Schmitt MP. 2019. Identification of zinc and Zur-regulated genes in Corynebacterium diphtheriae. PLoS ONE 14:e0221711. doi:10.1371/journal.pone.0221711 31454392 PMC6711530

[11] Peng ED, Lyman LR, Schmitt MP. 2021. Analysis of the manganese and MntR regulon in Corynebacterium diphtheriae. J Bacteriol 203:e0027421. doi:10.1128/JB.00274-21 34370555 PMC8459757

[12] Kunkle CA, Schmitt MP. 2003. Analysis of the Corynebacterium diphtheriae DtxR regulon: identification of a putative siderophore synthesis and transport system that is similar to the Yersinia high-pathogenicity island-encoded yersiniabactin synthesis and uptake system. J Bacteriol 185:6826–6840. doi:10.1128/JB.185.23.6826-6840.2003 14617647 PMC262719

[13] Bobrov AG, Kirillina O, Fetherston JD, Miller MC, Burlison JA, Perry RD. 2014. The Yersinia pestis siderophore, yersiniabactin, and the ZnuABC system both contribute to zinc acquisition and the development of lethal septicaemic plague in mice. Mol Microbiol 93:759–775. doi:10.1111/mmi.12693 24979062 PMC4132657

[14] Schneewind O, Mihaylova-Petkov D, Model P. 1993. Cell wall sorting signals in surface proteins of gram-positive bacteria. EMBO J 12:4803–4811. doi:10.1002/j.1460-2075.1993.tb06169.x 8223489 PMC413927

[15] Bailey TL, Elkan C. 1994. Fitting a mixture model by expectation maximization to discover motifs in biopolymers. Proc Int Conf Intell Syst Mol Biol 2:28–36. 7584402

[16] Jumper J, Evans R, Pritzel A, Green T, Figurnov M, Ronneberger O, Tunyasuvunakool K, Bates R, Žídek A, Potapenko A, et al.. 2021. Highly accurate protein structure prediction with AlphaFold. Nature 596:583–589. doi:10.1038/s41586-021-03819-2 34265844 PMC8371605

[17] Varadi M, Bertoni D, Magana P, Paramval U, Pidruchna I, Radhakrishnan M, et al.. 2023. AlphaFold Protein Structure Database in 2024: providing structure coverage for over 214 million protein sequences. Nucleic Acids Res 52:D368–D375. 10.1093/nar/gkad1011 PMC10767828 37933859

[18] Yatsunyk LA, Easton JA, Kim LR, Sugarbaker SA, Bennett B, Breece RM, Vorontsov II, Tierney DL, Crowder MW, Rosenzweig AC. 2008. Structure and metal binding properties of ZnuA, a periplasmic zinc transporter from Escherichia coli. J Biol Inorg Chem 13:271–288. doi:10.1007/s00775-007-0320-0 18027003 PMC2630496

[19] Yekwa EL, Serrano FA, Yukl E. 2022. Conformational flexibility in the zinc solute-binding protein ZnuA. Acta Crystallogr F Struct Biol Commun 78:128–134. doi:10.1107/S2053230X22001662 35234138 PMC8900738

[20] Schmitt MP. 2002. Analysis of a DtxR-like metalloregulatory protein, MntR, from Corynebacterium diphtheriae that controls expression of an ABC metal transporter by an Mn^2+^-dependent mechanism. J Bacteriol 184:6882–6892. doi:10.1128/JB.184.24.6882-6892.2002 12446639 PMC135481

[21] Lyman LR, Peng ED, Schmitt MP. 2021. The Corynebacterium diphtheriae HbpA hemoglobin-binding protein contains a domain that is critical for hemoprotein binding, cellular localization, and function. J Bacteriol 203:e0019621. doi:10.1128/JB.00196-21 34370560 PMC8508117

[22] Allen CE, Schmitt MP. 2009. HtaA is an iron-regulated hemin binding protein involved in the utilization of heme iron in Corynebacterium diphtheriae. J Bacteriol 191:2638–2648. doi:10.1128/JB.01784-08 19201805 PMC2668399

[23] Lyman LR, Peng ED, Schmitt MP. 2018. The Corynebacterium diphtheriae iron-regulated surface protein HbpA is involved in the utilization of the Hemoglobin-Haptoglobin complex as an iron source. J Bacteriol 200:e00676-17. doi:10.1128/JB.00676-17 29311283 PMC5847658

[24] Fischetti VA. 2019. Surface proteins on gram-positive bacteria. Microbiol Spectr 7. doi:10.1128/microbiolspec.gpp3-0012-2018 PMC6684298 31373270

[25] Swaminathan A, Mandlik A, Swierczynski A, Gaspar A, Das A, Ton-That H. 2007. Housekeeping sortase facilitates the cell wall anchoring of pilus polymers in Corynebacterium diphtheriae. Mol Microbiol 66:961–974. doi:10.1111/j.1365-2958.2007.05968.x 17919283 PMC2841690

[26] Ton-That H, Schneewind O. 2003. Assembly of pili on the surface of Corynebacterium diphtheriae. Mol Microbiol 50:1429–1438. doi:10.1046/j.1365-2958.2003.03782.x 14622427

[27] Ramirez NA, Das A, Ton-That H. 2020. New paradigms of pilus assembly mechanisms in gram-positive Actinobacteria. Trends Microbiol 28:999–1009. doi:10.1016/j.tim.2020.05.008 32499101 PMC7657965

[28] Bhat AH, Nguyen MT, Das A, Ton-That H. 2021. Anchoring surface proteins to the bacterial cell wall by sortase enzymes: how it started and what we know now. Curr Opin Microbiol 60:73–79. doi:10.1016/j.mib.2021.01.013 33611145 PMC7990056

[29] Gaspar AH, Ton-That H. 2006. Assembly of distinct pilus structures on the surface of Corynebacterium diphtheriae. J Bacteriol 188:1526–1533. doi:10.1128/JB.188.4.1526-1533.2006 16452436 PMC1367254

[30] Sue CK, Cheung NA, Mahoney BJ, McConnell SA, Scully JM, Fu JY, Chang C, Ton-That H, Loo JA, Clubb RT. 2024. The basal and major pilins in the Corynebacterium diphtheriae SpaA pilus adopt similar structures that competitively react with the pilin polymerase. Biopolymers 115:e23539. doi:10.1002/bip.23539 37227047 PMC11164409

[31] Peixoto RS, Antunes CA, Lourêdo LS, Viana VG, Santos C dos, Fuentes Ribeiro da Silva J, Hirata Jr. R, Hacker E, Mattos-Guaraldi AL, Burkovski A. 2017. Functional characterization of the collagen-binding protein DIP2093 and its influence on host–pathogen interaction and arthritogenic potential of Corynebacterium diphtheriae. Microbiology (Reading, Engl) 163:692–701. doi:10.1099/mic.0.000467 28535857

[32] Plumptre CD, Ogunniyi AD, Paton JC. 2012. Polyhistidine triad proteins of pathogenic Streptococci. Trends Microbiol 20:485–493. doi:10.1016/j.tim.2012.06.004 22819099

[33] Eijkelkamp BA, Pederick VG, Plumptre CD, Harvey RM, Hughes CE, Paton JC, McDevitt CA. 2016. The first histidine triad motif of PhtD is critical for zinc homeostasis in Streptococcus pneumoniae. Infect Immun 84:407–415. doi:10.1128/IAI.01082-15 26573735 PMC4730578

[34] Kunitomo E, Terao Y, Okamoto S, Rikimaru T, Hamada S, Kawabata S. 2008. Molecular and biological characterization of histidine triad protein in group A Streptococci. Microbes Infect 10:414–423. doi:10.1016/j.micinf.2008.01.003 18403236

[35] Loisel E, Chimalapati S, Bougault C, Imberty A, Gallet B, Di Guilmi AM, Brown J, Vernet T, Durmort C. 2011. Biochemical characterization of the histidine triad protein PhtD as a cell surface zinc-binding protein of Pneumococcus. Biochemistry 50:3551–3558. doi:10.1021/bi200012f 21425866

[36] Adamou JE, Heinrichs JH, Erwin AL, Walsh W, Gayle T, Dormitzer M, Dagan R, Brewah YA, Barren P, Lathigra R, Langermann S, Koenig S, Johnson S. 2001. Identification and characterization of a novel family of pneumococcal proteins that are protective against sepsis. Infect Immun 69:949–958. doi:10.1128/IAI.69.2.949-958.2001 11159990 PMC97974

[37] Li S, Liang H, Zhao S-H, Yang X-Y, Guo Z. 2023. Recent progress in pneumococcal protein vaccines. Front Immunol 14. doi:10.3389/fimmu.2023.1278346 PMC10560988 37818378

[38] Plumptre CD, Ogunniyi AD, Paton JC. 2013. Surface association of Pht proteins of Streptococcus pneumoniae. Infect Immun 81:3644–3651. doi:10.1128/IAI.00562-13 23876799 PMC3811752

[39] Sivaramalingam SS, Jothivel D, Govindarajan DK, Kadirvelu L, Sivaramakrishnan M, Chithiraiselvan DD, Kandaswamy K. 2024. Structural and functional insights of sortases and their interactions with antivirulence compounds. Curr Res Struct Biol 8:100152. doi:10.1016/j.crstbi.2024.100152 38989133 PMC11231552

[40] Popovic T, Kombarova SY, Reeves MW, Nakao H, Mazurova IK, Wharton M, Wachsmuth IK, Wenger JD. 1996. Molecular epidemiology of diphtheria in Russia, 1985-1994. J Infect Dis 174:1064–1072. doi:10.1093/infdis/174.5.1064 8896510

[41] Simon R, Priefer U, Pühler A. 1983. A broad host range mobilization system for in vivo genetic engineering: transposon mutagenesis in Gram negative bacteria. Nat Biotechnol 1:784–791. doi:10.1038/nbt1183-784

[42] Drazek ES, Hammack CA Sr, Schmitt MP. 2000. Corynebacterium diphtheriae genes required for acquisition of iron from haemin and haemoglobin are homologous to ABC haemin transporters. Mol Microbiol 36:68–84. doi:10.1046/j.1365-2958.2000.01818.x 10760164

[43] Cerdeño-Tárraga AM, Efstratiou A, Dover LG, Holden MTG, Pallen M, Bentley SD, Besra GS, Churcher C, James KD, De Zoysa A, et al.. 2003. The complete genome sequence and analysis of Corynebacterium diphtheriae NCTC13129. Nucleic Acids Res 31:6516–6523. doi:10.1093/nar/gkg874 14602910 PMC275568

[44] Schäfer A, Tauch A, Jäger W, Kalinowski J, Thierbach G, Pühler A. 1994. Small mobilizable multi-purpose cloning vectors derived from the Escherichia coli plasmids pK18 and pK19: selection of defined deletions in the chromosome of Corynebacterium glutamicum. Gene 145:69–73. doi:10.1016/0378-1119(94)90324-7 8045426

[45] Teufel F, Almagro Armenteros JJ, Johansen AR, Gíslason MH, Pihl SI, Tsirigos KD, Winther O, Brunak S, von Heijne G, Nielsen H. 2022. SignalP 6.0 predicts all five types of signal peptides using protein language models. Nat Biotechnol 40:1023–1025. doi:10.1038/s41587-021-01156-3 34980915 PMC9287161

[46] Sonnhammer EL, von Heijne G, Krogh A. 1998. A hidden Markov model for predicting transmembrane helices in protein sequences. Proc Int Conf Intell Syst Mol Biol 6:175–182. 9783223

[47] Krogh A, Larsson B, von Heijne G, Sonnhammer EL. 2001. Predicting transmembrane protein topology with a hidden Markov model: application to complete genomes. J Mol Biol 305:567–580. doi:10.1006/jmbi.2000.4315 11152613

[48] Sievers F, Wilm A, Dineen D, Gibson TJ, Karplus K, Li W, Lopez R, McWilliam H, Remmert M, Söding J, Thompson JD, Higgins DG. 2011. Fast, scalable generation of high-quality protein multiple sequence alignments using Clustal Omega. Mol Syst Biol 7:539. doi:10.1038/msb.2011.75 21988835 PMC3261699

