# Supplementary material for: Analysis of novel zinc-binding proteins in the cell wall of Corynebacterium diphtheriae
Source: J Bacteriol. 2025 Aug 18;207(9):e00239-25. doi: 10.1128/jb.00239-25 (PMC12445094; doi:10.1128/jb.00239-25)
Supplement: Figure S1 — Amino acid alignment. [file jb.00239-25-s0001.pdf]

Supplemental Material

```
ZnuE      GHIDIGAIEV-DGDFDLLA--------RD-----------------DTEAHPVWRHLDDMVFKVSDAALQTLPEDS---------TFEFTGAKAGDKVYVVPQSQI----SGVPWIGWNS  81
ZnuF 1    ------------GHFDALYGTYNY----SVKEPATDLPKL---ELMAVDGQQ-VVPQESLCFRLPPDAGEDGEELS-RIRIPDDEEFSF-LGKPGDIVWYAPQNIPFANGHRPIWAGIGA  98
ZnuF 2    GHMDLALTRP-DKEFVTVINREGK-----------------------------KYSSGDVTLAVPDSGGDQGGPLVKTDSWKDRIEAQI-SGSLPDQAWVLPESQD----HKLPWVGFSN  85
ZnuG      GHIDAFNVTAEDGGLKL--------TLKE-----------------DVTGQHVLHDPQDVILKVKQE--------------AMNEDVAK-VAEIGKPGYLLPMAQD----AGLIWPGWDT  76
CmrA2 1   -----------------------V----HVDSPNAFWDKKENNFILKSKSGD-VLPIEETVNWVSKGAKD--LGEY-VYRVPNDERLKF-LGEPGTRLYGAG--NPAGGKGTPIWAGFGA  86
CmrA2 2   GHVDIAPRED-GNELTLRLKDESGIAARE----------------------ATWREPKNVRFIVKED------RLV-----ALKNDTGL-LGNEGEKVYILPEDGRF--MSKHPWPGLST  83
CmrA2 3   GHLDFGPAFV-NDKLGFYIGDESGRTTDN-----------------TDASGHHVHDPSKVVLVVSPNRKRTLGKDV-----ELTEDTEF-IGKEGDTFYHLPLSED----HSAIWPGFDT  92
CmrA 1    THMDLSPEAAVTGANHNLEGEDDGDGGKEITGLGVVLGLRK--VNEETAEYK-GLPADNAVMWLPRS--------G-YYRFNSTEQTPF-VAKQGDTVWQAPQNVDA--SQLPIYFGYNS  105
CmrA 2    GHIDIRLAKN-NGKVGFNLNHEH-----------------------------VQRSLDDTIIRVRDNARQK-RDNK-----LGDEKWDF-LGPVGEKFYILPSSEE----PGKPWPGFSS  79
                                                                    .    :                           .      :               : * .

ZnuE      QAPS-IQ-------KVTDRGVTLELAGYQGPGHFSLFLQAGGVQKPQV--IWDA--DEKGAQPMWVELNTHTHANWVFTEPGVH  153
ZnuF 1    FDPHHEPKGKDSIPEHLDKNMYFELKDFSGPGDVNVFFKN-N-SRKEVERIFSS--NDEELKTIEYEMGSHGHFNWTFSKPGIY  178
ZnuF 2    EDLPSDA-------LLPDSKMTVSLNKVELPHDGRMISWHNGISGIDL--LTDT-DDL--SKNLEYGLHAHDHQSMLFTKEGAY  157
ZnuG      QGVR-EG-------G--FSAIDINFKKVSGPGE--VYMFKTGSFGGTESLLAGGSYELTSGSSIHQDPPSHVHTNWVFTEPGTY  148
CmrA2 1   DINLPTE-------KFRDGAFNMEIVDFKGPGKMELFRGT-GDPTDPVERFWSS--HEKGLRATWVDRGNHTHNQTTYTKPGQY  160
CmrA2 2   EELF-AK-------T--GKNYTFDFKTKSKPKNGAWMAFTGGNKVNPLKTIA----DSSKPASFKAEGSTHKHMSWGFTEPGIY  153
CmrA2 3   NKIA-HA-------F--PKGMDIEIKPESQPEGAQWYAHRFSNLGTAAERIA----DSMGPARIHNDGPFHSHLDWIFTKAGTY  162
CmrA 1    GSHIQKAAD---NEELQNQNYSLDLLKVEGPGDVEVFTQT-A---LRATRIFSS--KDKAFRSLIK--PRHSHYHTTFTKPGRY  178
CmrA 2    EDLNYKH-------Y--PQGVDITLDHAETPDGGRAVFYQADPLTGEVKRMLDTGD--EDTRTLSTTKPLHLHGNWSFNKPGQY  152
                                .   :   .   *                   :                   * *    :.: *   :
```

Figure S1. Amino acid sequence alignment for zinc protein domains.
Sequence alignment of amino acid sequences for putative zinc binding domains consisting of a conserved N-terminal HxD (green) and C-terminal HxH (green) with intervening sequence shown. The indicated Gly, Pro, and Gly residue (yellow) are also conserved.
